# Expression map of 78 brain-expressed mouse orphan GPCRs provides a translational resource for neuropsychiatric research

**DOI:** 10.1038/s42003-018-0106-7

**Published:** 2018-08-06

**Authors:** Aliza T. Ehrlich, Grégoire Maroteaux, Anne Robe, Lydie Venteo, Md. Taufiq Nasseef, Leon C. van Kempen, Naguib Mechawar, Gustavo Turecki, Emmanuel Darcq, Brigitte L. Kieffer

**Affiliations:** 1 IGBMC, Institut Génétique Biologie Moléculaire Cellulaire, Illkirch, France; 2 Douglas Mental Health University Institute and McGill University, Department of Psychiatry, Montreal, Canada; 3 Label Histologie, 51100 Reims, France; 4 Lady Davis Institute for Medical Research, Jewish General Hospital and McGill University, Department of Pathology, Montreal, Canada; 5 Present Address: INSERM U1151 Institut Necker Enfants Malades, 75014 Paris, France; 6 Present Address: Department of Pathology, Laboratory for Molecular Pathology, University Medical Centre Groningen, Groningen, The Netherlands

## Abstract

Orphan G-protein-coupled receptors (oGPCRs) possess untapped potential for drug discovery. In the brain, oGPCRs are generally expressed at low abundance and their function is understudied. Expression profiling is an essential step to position oGPCRs in brain function and disease, however public databases provide only partial information. Here, we fine-map expression of 78 brain-oGPCRs in the mouse, using customized probes in both standard and supersensitive in situ hybridization. Images are available at http://ogpcr-neuromap.douglas.qc.ca. This searchable database contains over 8000 coronal brain sections across 1350 slides, providing the first public mapping resource dedicated to oGPCRs. Analysis with public mouse (60 oGPCRs) and human (56 oGPCRs) genome-wide datasets identifies 25 oGPCRs with potential to address emotional and/or cognitive dimensions of psychiatric conditions. We probe their expression in postmortem human brains using nanoString, and included data in the resource. Correlating human with mouse datasets reveals excellent suitability of mouse models for oGPCRs in neuropsychiatric research.

## Abstract (web summary)

Aliza Ehrlich et al. report the fine-mapping of orphan GPCR (oGPCR) transcripts in the mouse brain using in situ hybridization and provide a public resource for data mining. The authors also mapped 25 selected oGPCRs in human brains, identifying oGPCRs with high correlation between species and potential roles in neuropsychiatric disorders.

## Introduction

G protein-coupled receptors (GPCRs) represent the largest receptor family for drug development in medicine (see GPCR database at http://www.guidetopharmacology.org/)^1^. As of November 2017, the 475 FDA approved drugs, which activate or block GPCRs, account for nearly 30% of all pharmaceuticals in current use^2–4^. These drugs in fact target only 27% of known GPCRs, with aminergic (dopamine, serotonin), cannabinoid and opioid receptors being most prominent targets for the central nervous system^3^. About 400 non-odorant GPCR genes have been identified in the genome, among which a subset of approximately 130 remain orphan GPCRs (oGPCRs), meaning that their endogenous ligand has not been identified^1,3–6^. Importantly, almost half of oGPCRs are expressed in the brain^3,7,8^ and, as are orphan neuropeptides^9^, each neural oGPCR represents an unprecedented opportunity to address brain function and disease^10,11^.

All GPCRs, including oGPCRs, are prime drug targets, as these receptors are easily accessible at the cell surface, and recent drug design strategies utilize allostery, bias or structure-based docking approaches to create new drugs^12–14^. Hence oGPCRs, in principle, have strong potential for drug design, however their function in the brain is poorly understood and, overall, these receptors are understudied^15^. In a few cases only, oGPCR genes are linked to a disease^3^, surrogate ligands have been developed (for a recent example see ref. ^16^), or a phenotype is reported after gene knockout and/or overexpression in mice (reviewed in the ref. ^5,17^), but overall the potential of oGPCRs for neuroscience and neuropsychiatry has not been systematically explored.

An essential first step toward this goal is to establish the expression pattern of oGPCR transcripts throughout the brain. Notable is the case of *GPR88*, whose striatal-enriched distribution described a decade ago^18–21^ attracted attention in both academia and industry. The *Gpr88* gene deletion in mice revealed multiple roles in behaviors related to striatal^22–26^ and sensory cortical^22,27,28^ functions with potential implications for both neurological and psychiatric disorders. Drug discovery efforts show very recent success for GPR88 agonist development^29,30^, and a first human genetic study reported association between *GPR88* variants and brain pathology, including learning and movement deficits^31,32^. The case of *GPR88*, or the example of GPR52^33,34^ demonstrate that elucidating oGPCR brain expression profiles is paramount to recognize the potential therapeutic value for any given oGPCR.

Two publicly available databases, which cover the entire genome, report microarray-based gene expression profiling of selected mouse (http://brainstars.org/multistate/)^35^, and human (Allen Institute, http://human.brain-map.org/) brain regions^36^. Mining for oGPCR expression in these resources is possible, although many oGPCRs remain below detection thresholds and the anatomical precision is poor. Two other sources of information are available, which report spatial transcript distribution for thousands of genes in the mouse brain using in situ hybridization (ISH) (Allen Institute 20,000 genes see ref. ^37^ and GENSAT 5000 genes see ref. ^38^), but in these approaches low sensitivity and high throughput strategies often hampers the detection of low abundant transcripts, as is the case for most oGPCRs. A single study described the distribution of all known GPCR transcripts using qPCR in samples from mouse tissues, including essentially peripheral tissues and some brain regions^7^. In this case there was no specific focus on orphan GPCRs, and their spatial distribution in the brain.

To tackle brain oGPCR anatomy in the brain, we fine-map their expression in the mouse brain using dedicated probes in two complementary in situ hybridization (ISH) approaches, and create a database of all images, posted as an open-access resource at http://ogpcr-neuromap.douglas.qc.ca. Further, we correlate oGPCR expression scores with data from the two most comprehensive public databases (mouse http://brainstars.org/multistate/ and human http://human.brain-map.org/) for cross-validation and to probe cross-species information. These analyses guide further selection of an oGPCR subgroup with expression in key brain centers for cognition, motivational drive and emotional processing, which we test in postmortem human brain samples (also in the database) to evaluate appropriateness of mouse models and the potential to address oGPCR contributions in neuropsychiatric disorders.

## Results

### The oGPCR-neuromap database

**Fig. 1:**
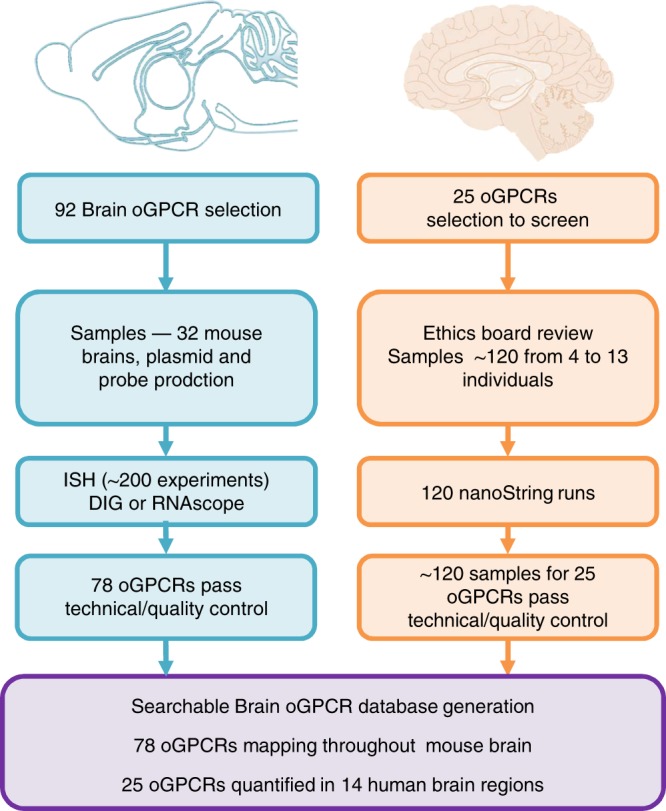
Generation of the oGPCR-neuromap. 92 brain oGPCRs were selected from a previous study reporting GPCR tissue distribution in mice^7^. Coronal sections were prepared from 32 mouse brains, and customized probes were generated by plasmid production and probe transcription for DIG in situ hybridization (ISH), and by the manufacturer for RNAscope ISH. For both approaches, 50 sections across 9 slides were assayed for each brain oGPCR, representing ~200 ISH experiments in total. 78 oGPCRs were merited for semi-quantitative analysis (Fig. 2). For these 78 oGPCRs, ~160 experiments containing 1350 slides of 8000 sections are deposited online in a searchable website http://ogpcr-neuromap.douglas.qc.ca. 25 oGPCRs were selected (Supplementary Table 2) for profiling in the human brain. Approval was obtained from the institutional review board. Roughly 120 human samples were prepared from 4 to 13 postmortem individuals to span 14 brain regions, used for RNA preparation and quality controlled. Customized nanoString probes were designed to target the 25 oGPCR transcripts, which were quantified in all the 120 samples. Raw data from each individual sample are also deposited in the oGPCR-neuromap resource

The process for creating the oGPCR database is summarized in Fig. 1. Initial information came from a previous qPCR-based expression study of non-odorant GPCRs in central and peripheral adult mouse tissues^7^. From this report, we compiled a list of 92 oGPCRs that were detected in the brain, among which about 50% where present in brain only. Mouse brains were sectioned coronally and processed with customized probes to target brain oGPCRs in both digoxigenin (DIG)-ISH (Supplementary Data 1) and RNAscope ISH experiments. Control probes were included in all the ISH experiments (Supplementary Figure 1a) and about 50 brain sections spanning the brain were labeled for each probe. All receptors were studied, however data for only 78 oGPCRs are shown and discussed here. We eliminated a few candidates when technical problems impeded ISH image analysis. In the final collective, 51 oGPCRs are rhodopsin-like (class A), 18 oGPCRs belong to the adhesion family (class B), 3 are members of the glutamate family (class C), and 6 oGPCRs belong to other classes such as frizzled^5,39–41^. A selection of 25 oGPCRs (see below) was further studied in 120 samples from 4 to 13 adult human individuals, including 14 brain regions and using custom-made probes by digital gene expression nanoString technology.

Overall, image datasets from almost 160 experiments, on over 8000 coronal mouse brain sections across 1350 slides are uploaded in the database. Original slide scanner images can be searched by gene name or technique as well as representative images for control probes for each technique, DIG-ISH or RNAscope ISH. The human individual subject data are also deposited in the resource. This open access resource is now available to researchers and clinicians online at http://ogpcr-neuromap.douglas.qc.ca.

### Clustering mouse oGPCR expression levels using DIG-ISH data

**Fig. 2:**
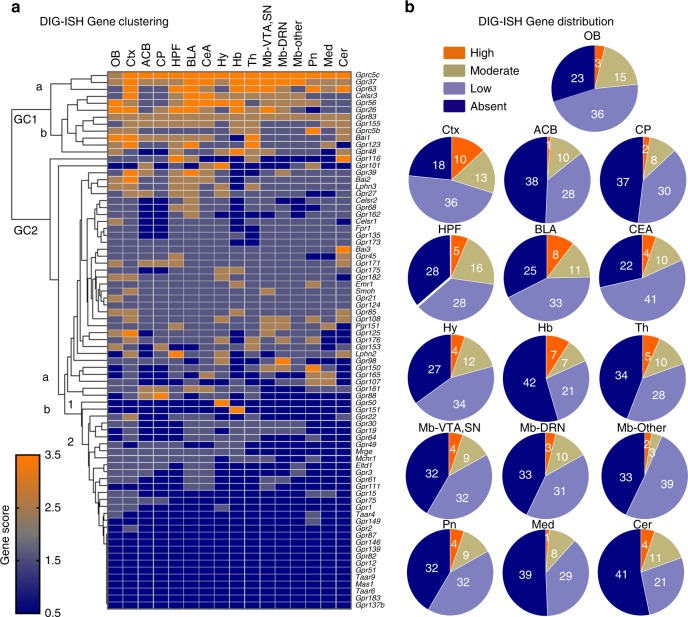
Clustering oGPCR distribution in the mouse brain. **a**, **b** Semi quantification of DIG-ISH mapping of oGPCRs in the adult mouse brain. **a** Heatmap showing hierarchical clustered distribution of oGPCRs (*Gpr17* was not scored due to unvaried expression) mapped by scoring ISH images across 16 brain regions in the mouse. Gene cluster 1 (GC1) principally includes widespread oGPCRs with strong (GC1a) or moderate (GC1b) expression across the brain. Gene cluster 2 (GC2) essentially contains localized oGPCRs with moderate to high (GC2a and b1) or widespread low expression patterns (GC2a and b2) as well as absent oGPCRs (GC2b2). Color bar indicate 4 scoring levels of expression high (3.5, orange), moderate (2.5, brown), low (1.5, greyish blue) or absent (0.5, blue). **b** Pie charts illustrate oGPCR distribution (high/moderate/low/absent) according to the brain region (See Supplementary Data 2 for oGPCR listings). Annotated brain regions: OB, olfactory bulb; Ctx, cortex; ACB, nucleus accumbens; CP, caudate putamen; HPF, hippocampal formation; BLA, basal-lateral amygdala; CeA, central extended amygdala; Hy, hypothalamus; Hb, habenula;, Th, thalamus; Mb, midbrain; VTA, ventral tegmental area; SN, substantia nigra; DRN, dorsal raphe nucleus; Mb-other (general midbrain excluding aforementioned areas); Pn, pons; Med, medulla; Cer, cerebellum

To further exploit the mouse DIG-ISH resource, we semi-quantified the ISH signal for each oGPCR by manual observation, and data from two independent observers were compared. Discrepancy was rare, but in this case data were confronted and agreed for consistency. Scoring was performed across 16 regions selected to span the entire brain. Scoring for each oGPCR was done on a scale of 4 levels of expression high (3.5), moderate (2.5), low (1.5), and absent (0.5). As labeling intensities may differ between probes, scoring was performed based on relative intensities across all brain sections for each probe dataset, in a within design. As an example, the striatal receptor *Gpr88* was absent in olfactory bulb (OB), low in cortex (Ctx), moderate in nucleus accumbens (ACB) and high in caudate putamen (CP) (Supplementary Figure 1b, top), in agreement with previous reports^20–22,28,42^. Unsupervised hierarchical gene clustering of the DIG-ISH scoring revealed 2 principal gene cluster nodes, GC1 and GC2, the former containing oGPCRs with widespread distribution and the latter containing those with highly restricted, low or undetectable expression (Fig. 2a).

GC1 is primarily composed of genes with broad expression throughout mid- to forebrain and contains 2 groups. GC1a includes *Gprc5c*, *Gpr37*, and *Gpr63*, all showing moderate to high expression in Ctx, basolateral amygdala (BLA), habenula (Hb), thalamus (Th), and midbrain (Mb)—ventral tegmental area (VTA)/substantia nigra (SN). GC1b includes 3 clusters with high expression in BLA (*Celsr3*, *Gpr56*, and *Gpr26)*, or in Ctx and Th (*Gpr123*, *Bai1*), and moderate expression across Ctx, Hb, Th, and cerebellum (Cer) (*Gpr83, Gpr155*, and *Gprc5b*). *Gpr48* is isolated displaying highest expression in Hb.

More localized oGPCRs were found throughout the GC2 node. Prominent clusters in GC2a are as follows: *Gpr88* and *Gpr161* form a small cluster, sharing expression in ACB and central extended amygdala (CeA). Additionally, *Gpr107*, *Gpr165*, and *Gpr150* are expressed in medulla (Med) and pons (Pn), a region containing noradrenergic nuclei (locus coeruleus). Next, adhesion receptor *Gpr98* shows high expression in the serotonergic dorsal raphe nucleus (DRN). Lastly, a BLA cluster (*Celsr2 Gpr68*, *Gpr162*) is adjacent to cortico-hippocampal and amygdalar oGPCRs (*Gpr27*, *Gpr39*, *Lphn3*, *Bai2)*. In GC2b1, 2 oGPCRs show remarkably localized expression, *Gpr50* only in Hy and *Gpr151* highly enriched in the Hb. The last subgroup GC2b2, consists of 27 oGPCRs, 16 with low expression, the exception being *Gpr22* with moderate expression in Ctx, and 11 oGPCRs were undetectable by this ISH method.

We examined the distribution of oGPCRs across the four categories (high/moderate/low/absent) in the 16 brain regions (Fig. 2b). Depending on the brain region 45–77% oGPCRs were detected in each region and, notably, OB, Ctx, HPF, BLA, and CeA expressed a large part (49–59) of the entire oGPCR group (see oGPCR details in Supplementary Data 2). We searched for potential cell types expressing the 78 oGPCRs using the public RNA-Seq database of adult mouse cortical purified cells (https://web.stanford.edu/group/barres_lab/brain_rnaseq.html)^43^ and found 21 neuronal, 10 in astrocytes, 10 microglial, 8 endothelial, 11 oligodendrocytic oGPCRs, while 17 oGPCRs were virtually undetectable in this database (Table 1). In conclusion, the semi-quantification demonstrates highly distinguishable expression patterns, ranging from ubiquitous to very localized, and points at a number of oGPCRs with highly spatially restricted distribution that may be indicative of specialized brain functions.

**Table 1:** oGPCR cell subtype

Neurons	*Celsr2, Celsr3, Gpr12, Gpr21, Gpr22, Gpr26, Gpr27, Gpr45, Gpr61, Gpr64, Gpr83, Gpr85, Gpr88, Gpr123, Gpr135, Gpr149, Gpr151, Gpr161, Gpr162, Gpr173,* and *Mchr1*
Astrocytes	*Bai2, Bai3, Celsr1, Gpr3, Gpr19, Gpr48, Gpr51, Gpr63, Gpr98, Smoh*
Microglia	*Emr1, Fpr1, Gpr56, Gpr107, Gpr108, Gpr137b, Gpr153, Gpr165, Gpr175, Gpr183*
New oligodendrocytes	*Gpr15, Gpr17, Gpr155, Gpr176*
Myelinating oligodendrocytes	*Gpr37, Gprc5b*
OPCs	*Bai1, Gpr49, Gpr75, Gpr125, Lphn3, Mrge*
Endothelial	*Eltd1, Gpr30, Gpr116, Gpr124, Gpr146, Gpr182, Gprc5c, Lphn2*
Virtually undetectable	*Gpr1, Gpr2, Gpr39, Gpr50, Gpr68, Gpr82, Gpr87, Gpr101, Gpr111, Gpr139, Gpr150, Gpr171, Mas1, Pgr15l, Taar4, Taar9, Taar6*

RNA-Seq database of purified neurons, astrocytes, microglia, endothelial cells, pericytes, and various maturation states of oligodendrocytes from mouse cortices reveal the cell subtype of indicated oGPCRs (https://web.stanford.edu/group/barres_lab/brain_rnaseq.html)^43^. For clarity, each oGPCR is shown in the category with the highest Fragments Per Kilobase of transcript per Million (FPKM) reads, and is classified as virtually undetectable when FPKM is below 1.0

### Detecting additional oGPCRs with supersensitive RNAscope ISH

Not all the oGPCR transcripts could be detected by DIG-ISH (see Fig. 2a, GC2b2). Since GPCRs are notoriously difficult to detect because of overlapping sequence homology and low expression, we improved our chances of successful oGPCR detection by repeating the entire mapping experiment using another ISH approach. RNAscope is a highly sensitive ISH method that robustly amplifies the signal of individual RNA molecules with no cross-hybridization^44^. In addition, using a second method would confirm findings from the standard DIG-ISH experiment.

For all oGPCRs dedicated probes were designed and control probes were included in each experiment (Supplementary Fig. 1a, bottom). For the majority of oGPCRs in this study, detection was achieved at a similar level to DIG-ISH as shown for *Gpr88* (Supplementary Fig. 1b, bottom) *and Gpr50*, absent in PFC and ACB but remarkable in Hy (compare *Gpr50* in Hy in Fig. 2a and Supplementary Fig. 2). For some oGPCRs, RNAscope increased regional identification, as seen with *Gpr68* in PFC, CP, and ACB, which was low or absent with DIG-ISH (compare Fig. 2a and Supplementary Fig. 2). For the 16 oGPCRs with only low detection in DIG-ISH, 11 oGPCRs had improved detection with RNAscope. These included, *Gpr139* highly expressed in PFC, CP and MHb, *Gpr149* with moderate to low expression in ACB, CeA and VTA, and *Gpr162* was highly detectable in PFC moderate in CP and low in VTA (Supplementary Fig. 2).

Thus, the two distinct ISH methods yielded comparable distribution of oGPCR expression, cross-validating our results, and added highly sensitive detection for low abundant oGPCRs.

### Correlating ISH data with public mouse and human datasets

**Fig. 3:**
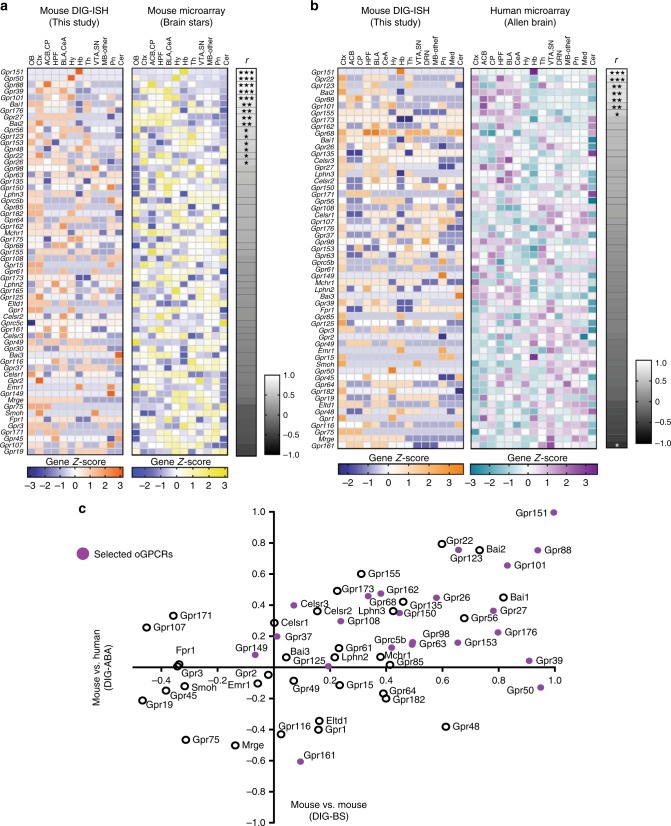
Correlation analyses of DIG-ISH data with public genome-wide microarray datasets. **a** Expression profiles for 60 oGPCR in 12 mouse brain regions, found in both mouse DIG-ISH data from this study (Left panel orange (high) to dark blue (low) and Fig. 2a) and mouse microarray data fro the BrainStar platform (http://brainstars.org/ BrainStars, Riken, Japan)^35^ (middle panel, yellow (high) to light blue (low)). **b**, Expression profiles for 56 oGPCRs in 15 brain regions, found in both DIG-ISH from this study (left, orange (high) to dark blue (low) color scale) and Allen Brain’s human data (middle, magenta (high) to cyan (low) color scale). **a**, **b**, Clustering shows best similar (top) to less-well correlated (bottom) oGPCR expression patterns. To the *right* of each oGPCR comparison, a grayscale gradient shows Pearson correlation coefficients (*r*) from positive (*white*) to inverse (*black*) correlation coefficients, scale −1 to 1.0. oGPCRs with statistically significant similar correlation coefficients are indicated as ***P < 0.001, **P < 0.01, *P < 0.05. Except for *Gpr68* in **b**, no gene profile comparison showed significant difference according to students *t*-tests (Supplementary Table 1). OB, olfactory bulb; Ctx, cortex; ACB, nucleus accumbens; CP, caudate putamen; HPF, hippocampal formation; BLA, basal-lateral amygdala; CeA, central extended amygdala; Hy, hypothalamus; Hb, habenula; Th, thalamus; VTA, ventral tegmental area; SN, substantia nigra; Mb-other (general midbrain excluding aforementioned areas), Pn, pons; Cer, cerebellum. oGPCRs were excluded from analysis if the regional expression did not vary or the oGPCR was not found in the public dataset. **c** Correlation between Pearson coefficients for 56 shared oGPCRs in **a**, **b** is shown. The majority of oGPCRs (34) show positive correlations in both datasets (top right quadrant). Purple filled circles represent 23 of the 25 selected oGPCRs (Supplementary Table 2) for nanoString analysis in human brain samples. *Gpr139* and *Gprc5c* were not available for correlation. Pearson correlation coefficient was statistically significant, *r* was 0.4987, ***P-value < 0.0001

Next, we converted the semi-quantified ISH data into *Z*-scores (see methods) to compare data from this study with available datasets. A first mouse-mouse data comparison was important for validation. We searched for publicly available mouse transcriptome databases, and selected the BrainStars platform (http://brainstars.org/) having the highest number of oGPCRs. We retrieved the DNA-microarray data obtained from microdissected mouse brain samples^35^, and performed correlation analyses for each gene. We first tested the extent to which expression profiles differ between the two mouse datasets using Student’s *t*-tests (Supplementary Table 1) and found that distribution profiles for 60 oGPCRs across 12 brain regions in the two datasets were not statistically different. We then probed similarities between expression patterns using Pearson correlation analysis and found that 80% of oGPCRs display positive Pearson coefficients (*r*), among which nearly half (31%) show statistically significant similarity (Pearson correlation, *r* from 0.5788 to 0.99609*, P* values from 0.0486 to *<*0.0001 also see Supplementary Table 3) (Fig. 3a). Only 20% of oGPCRs showed inversely correlated expression patterns, and these trends remained non-significant (*P* *>* 0.05). Overall therefore, the two mouse datasets showed highly comparable distribution of oGPCR expression.

A mouse–human data comparison is a further critical step in the context of drug development and the relevance of animal models. For cross-species analysis, we compared 56 oGPCR genes present in the human DNA-microarray results from Allen Brain (http://human.brain-map.org/36) with mouse DIG-ISH data from this study, and across 15 brain regions. With the exception of *Gpr68*, oGPCR expression profiles did not differ statistically using the Student’s *t*-test (Supplementary Table 1). Further, Pearson correlation analysis indicated that 70% of oGPCRs showed positively correlated expression patterns, of which 18% showed statistical significance between our own and human data (Pearson correlation, *r* from 0.6016 to 0.9964*, P* values from 0.0165 to *<*0.0001 also see Supplementary Table 3 and Fig. 3b). Otherwise 30% of oGPCRs were inversely correlated, with only *Gpr161* reaching statistical significance. Together, and as expected, cross-species comparison shows less similarity than within-species comparison, and suggests that expression patterns of *Gpr68* and *Gpr161* in particular may largely differ across mouse and human.

We finally identified oGPCRs with high similarity in transcript distribution both within and across species, by combining mouse-mouse and mouse-human correlations in a new Pearson correlation analysis. The overall comparison yielded a Pearson coefficient that was statistically significant (Pearson correlation *r* = 0.4987, *P* = 0.000092, 95% CI 0.2714–0.6733) and highlighted a number of oGPCRs commonly found in both mouse–mouse and mouse–human data (Fig. 3c). In conclusion, correlation analyses identified 34 oGPCRs whose expression profile in our study correlates well with existing data, an information that extends in-depth oGPCR fine-mapping of the present resource.

### Focusing on 25 oGPCRs and mouse brain function

To exemplify the potential of the oGPCR-neuromap for target discovery in the area of neuropsychiatric diseases, we next selected a sub-group of 25 oGPCR transcripts, to be examined further in the human brain. Criteria were as follows, localized rather than widespread expression for their potential to have restricted over wide-ranging functions (Supplementary Table 2, Column II), high correlation coefficients between mouse DIG-ISH and BrainStars (Supplementary Table 2, Column III; Fig. 3a), high correlation coefficients between mouse DIG-ISH and human Allen brain (Supplementary Table 2, Column IV; Fig. 3b) and low most number of existing original publications based on PubMed records (Supplementary Table 2, Column V). The table identified 25 oGPCRs fitting our criteria (Supplementary Table 2 oGPCRs in gold; Fig. 3c in purple). In this collection of 18 Class A, 4 Class B, 2 Class C and a single oGPCR classified as other (Supplementary Table 2) were of potential mouse to human translational relevance, and interest for brain disorders.

**Fig. 4:**
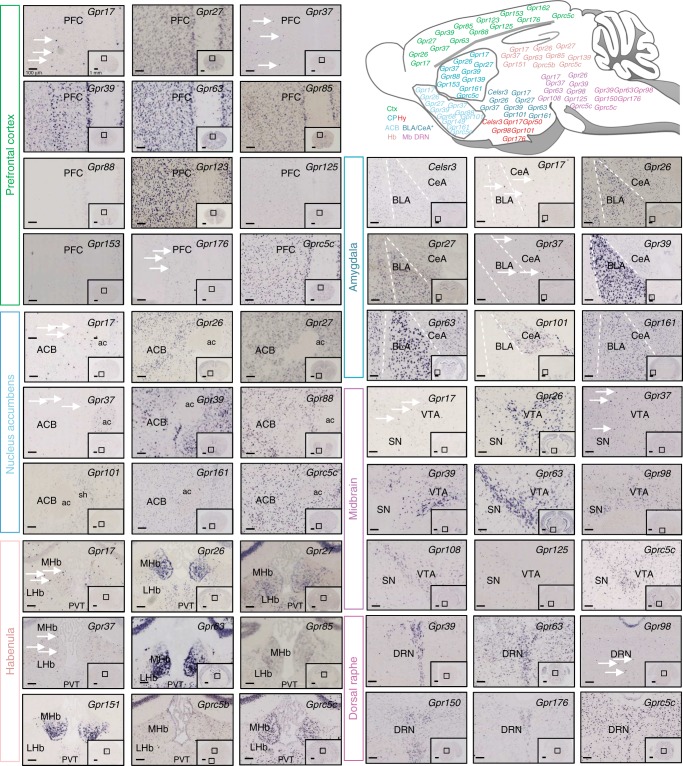
oGPCRs in brain centers relevant to cognition, motivational drive and emotional processing. Top right, a sagittal scheme shows high expression sites for the 25 oGPCR subselection. A color is assigned to each brain region, and ISH image panels show expression patterns of remarkable oGPCRs in these regions (see Supplementary Figure 3 for OFC, CP, and Hy). Top left panel, prefrontal cortex (PFC), critical for cognition, reward learning and inhibitory controls, expresses *Gpr17, Gpr27*, *Gpr37*, *Gpr39*, *Gpr63*, *Gpr85*, *Gpr88*, *Gpr123 (Adgra1)*, *Gpr125 (Adgra3)*, *Gpr153*, *Gpr176*, and *Gprc5c.* Middle left panel, oGPCR expressed in the nucleus accumbens (ACB), a key center for reward and motivation, are *Gpr17*, *Gpr26*, *Gpr27*, *Gpr37*, *Gpr39*, *Gpr88*, *Gpr101*, *Gpr161*, and *Gprc5c*. Bottom left panel, habenula (Hb), an area critical for reward valuation, aversive processing and decision-making shows *Gpr17*, *Gpr26*, *Gpr27*, *Gpr37*, *Gpr63*, *Gpr85*, *Gpr151*, *Gprc5b*, and *Gprc5c* expression. *Top right* panel, amygdala, a major center processing fear and negative affect, expresses *Celsr3* (*Adgrc3*), *Gpr17*, *Gpr26*, *Gpr27*, *Gpr37*, *Gpr39*, *Gpr63*, *Gpr101*, and *Gpr161*. The basal lateral amygdala (BLA) is delimited by a white dashed line from the central extended amygdala (CeA). Middle right panel, oGPCRs expressed in midbrain dopaminergic nuclei (VTA, ventral tegmental area; SN, substantia nigra) central for movement and reward-related behaviors, are *Gpr17*, *Gpr26*, *Gpr37*, *Gpr39*, *Gpr63*, *Gpr98 (Adgrv1)*, *Gpr108*, *Gpr125 (Adgra3)*, and *Gprc5c. Bottom right* panel, the serotonergic dorsal raphe nuclei (DRN), a main brain center for emotional responses and depressive states, shows expression of *Gpr39*, *Gpr63*, *Gpr98*, *Gpr150*, *Gpr176*, and *Gprc5c*. The 1.25× insets show whole ection view at Allen brain atlas levels #32-40 (PFC), #43-47 (ACB), #65-71 (Hb), #68-75 (BLA/CeA), #81-89 (Mb-VTA/SN), and #98-105 (DRN) with a black box outlining the corresponding magnified area. Scale bar for 1.25× is 1 mm and 10× is 100 µm. White arrows demonstrate sparse DIG labeling pattern for *Gpr17*, *Gpr37*, *Gpr39*, *Gpr98*, and *Gpr176*. Ctx, Cortex; CP, caudate putamen; Mb, midbrain; Hy, hypothalamus; ac, anterior commissure; sh, shell; MHb, medial habenula; LHb, lateral habenula; PVT, paraventricular thalamus; DRN, dorsal raphe nucleus

We next mined the oGPCR-neuromap resource for these 25 oGPCRs, in order to extract mapping information, with a focus on brain centers that govern cognition, motivational drive and emotional processing, whose deregulation is known to cross-cut most psychiatric symptoms (Fig. 4, top right) and are highly studied in preclinical neuroscience research^45–48^. A selection of sections is shown in Fig. 4 with most remarkable expression patterns, and the potential relevance to the disease areas of addiction and depression are detailed below as an example.

Cortical areas involved in decision-making and inhibitory controls, particularly in relation to reward learning and emotional experiences, include the orbital (OFC) and prefrontal (PFC) cortices^45,49^, and their function is heavily impaired in both addiction^50^ and depression^51^. OFC shows sparse patterns *for Gpr17*, *Gpr37*, *Gpr39*, and *Gpr125 (Adgra3*), whereas *Gpr26*, *Gpr63*, *Gpr85*, *Gpr123 (Adgra1)*, and *Gprc5c* are dense throughout this brain region (Supplementary Figure 3, *top panel*). Meanwhile the nearby PFC (Fig. 4, top left) contains sparse distribution pattern for *Gpr17*, *Gpr37*, and *Gpr176*. Some prefrontal oGPCR transcripts show a cortical layer pattern, *Gpr88* and *Gpr153*, while others appear broadly expressed across cortical layers, i.e., *Gpr27*, *Gpr39*, *Gpr63*, *Gpr85*, *Gpr123 (Adgra1), Gpr125* (Adgra3), and *Gprc5c*.

The striatum is composed of the dorsal CP and the ventral ACB, which are main projection sites for dopamine neurons from SN and VTA, respectively, and fulfill different functions. In the CP, which controls motor responses and is also involved in compulsive-like behaviors characterizing drug abuse^52^, we find *Gpr17*, *Gpr37*, *Gpr39*, *and Gpr153* are sparsely localized while *Gpr26*, *Gpr27*, *Gpr88*, *Gpr161*, and *Gprc5c* are broadly expressed throughout the region (Supplementary Figure 3, *middle*). In the ACB, the main center for reward and motivated behaviors^53,54^, *Gpr17 and Gpr37* feature a sparse pattern whereas *Gpr26*, *Gpr27*, *Gpr39*, *Gpr88*, *Gpr161*, and *Gprc5c* are more widely distributed (Fig. 4, middle left). Remarkably, *Gpr101* not seen in the dorsal striatum is found to be restricted in the shell of the ACB, a compartment implicated in both drug and food reward^46,55^.

The habenula is an epithalamic structure composed of a lateral (LHb) and medial (MHb) part, which has attracted increasing attention in both areas of addiction and depression, for a role in the anticipation of aversive outcomes (LHb, see Proulx et al.^56^ and Bromberg-Martin et al.^57^) and more generally for mediating aversive states^57–59^. Receptors in the MHb with a sparse pattern include *Gpr17* and *Gpr37* whereas *Gpr26*, *Gpr27*, *Gpr63*, *Gpr85*, *Gpr151*, *Gprc5b*, and *Gprc5c* exhibit a dense expression pattern (Fig. 4, bottom left). Of interest, *Gpr151* was not detected in any other brain region.

The hypothalamus, an area with accumbal inputs and outputs to VTA, directs food reward, motivation, and stress response via hypothalamic pituitary axis (HPA)^46,60^. Neurobiological adaptations to stress are now well-accepted environmental triggers of depression and addiction and GPCRs, such as CRF, which modulate those responses are emerging pharmacological targets. The hypothalamic oGPCR panel shows a sparse pattern for *Gpr17*, *Gpr50* and *Gpr98* (Supplementary Figure 3, bottom). Whereas, *Celsr3 (Adgrc3)*, *Gpr101 and Gpr176* are densely localized throughout several hypothalamic nuclei. Interestingly, around the third ventricle a dense layer of cells feature labeling for only *Gpr50* and *Gpr98*. Notably, *Gpr50* was only detectable in this region of the brain.

Long hailed as the primary seat of emotional responses, fear and anxiety, the amygdala, is composed of several subnuclei with diverse functions, BLA having roles in directing both negative and positive valence and the CeA primarily involved in the negative responses to fearful, stressful and drug-related stimuli^61–64^. DIG-ISH shows *Gpr17* and Gpr37 are expressed sparingly in the neighboring BLA and CeA and *Celsr3* (*Adgrc3*), *Gpr26*, *Gpr161* show even distribution across the amygdala (Fig. 4, top right). Remarkably, *Gpr27*, *Gpr39* and *Gpr63* show enriched expression in the BLA whereas *Gpr101* is enriched in the CeA.

The hind-midbrain houses several monoaminergic-rich nuclei. Among them, VTA and SN are the major nuclei for dopaminergic neurons that project widely to the forebrain and mainly control motor activity (SN), reward and motivation (SN and VTA)^48,65^. While SN neuronal loss is a main feature of Parkinson’s and Huntington’s diseases^66–68^, VTA dysfunction strongly impairs hedonic homeostasis and motivation, a hallmark of both addiction and depression^46,47^. The midbrain panel shows *Gpr26*, *Gpr39* and *Gpr98 (Adgrv1), Gpr63*, *Gpr108*, *Gpr125 (Adgra3)* and *Gprc5c* are found throughout the SN and VTA though *Gpr17* and *Gpr37* are parsimoniously expressed (Fig. 4, *middle right*). The serotonin-rich dorsal raphe (DRN) rich, is another monoaminergic nucleus in the midbrain. Serotonin is rewarding as it regulates mood and drugs that increase it act as anti-depressants^46,69,70^. Therefore, dorsal raphe oGPCRs are probable targets for dysfunctional reward systems and mood disorders. Brain mapped oGPCRs enriched in the raphe show dense localization for *Gpr39*, *Gpr63*, *Gpr150*, *Gpr176*, *Gprc5c* and sparse cell pattern for *Gpr98* (Fig. 4, bottom right).

Expression datasets are currently available for these 25 selected oGPCRs in public resources. We further compared our ISH mapping dataset with three mouse databases, including one qPCR dataset^7^ [https://kidbdev.med.unc.edu/databases/ShaunCell/home.php] and two ISH resources (GENSAT^38^ [http://www.gensat.org/bgem_ish.jsp] and Allen brain^37^ [http://mouse.brain-map.org/search/index]). Correlation analysis revealed significant positive Pearson correlations for 6 out of 25 (24%) for the qPCR dataset, 7 out of 10 (70%) for the GENSAT dataset and 10 out of 22 (45%) for the Allen brain dataset (Supplementary Fig. 4 and 5). In this comparison, datasets with the least similarity were also the most technically different (ISH vs. qPCR), confirming the critical advantage of the ISH resource we have created here that integrates two independent ISH analyses.

We next proceeded to examine the above selected oGPCRs in human postmortem brain tissue, using a highly sensitive method for gene expression analysis.

### Focusing on these 25 oGPCRs in the human brain

The nanoString digital multiplex nCounter assay^71^ directly amplifies each gene by a unique barcode permitting sensitive and reliable quantification as demonstrated by highly positive correlation of technical replicates (Pearson *r* = 0.9984, *P* < 0.0001, 95% CI 0.9964–0.9993; Supplementary Fig. 6, and see Methods). Human brain tissue was obtained from the Douglas Brain Bank http://douglasbrainbank.ca/ and included 4–13 individual subjects that had died suddenly from accidental or natural causes. To the best extent possible, we dissected brain areas corresponding to mouse brain areas of interest in this study. Samples were obtained from 14 different brain regions: orbital frontal cortex (OFC; BA11, *n* = 7), prefrontal cortex (PFC; BA9-10, *n* = 9), motor cortex (MoCtx; BA4, *n* = 9), somatosensory cortex (SSCtx; BA1, 2, 3, *n* = 9), nucleus accumbens (ACB, *n* = 9), caudate putamen (CP *n* = 13), habenula (Hb, *n* = 4), thalamus (Th + Hb, *n* = 9), Medulla (Med, *n* = 7), substantia nigra (SN, *n* = 7), Pons (Pn, *n* = 13) midbrain (Mb, *n* = 6), ventral tegmental area (VTA, *n* = 6) and cerebellum (Cer, *n* = 9). Data from this experiment are also available at http://ogpcr-neuromap.douglas.qc.ca.

**Fig. 5:**
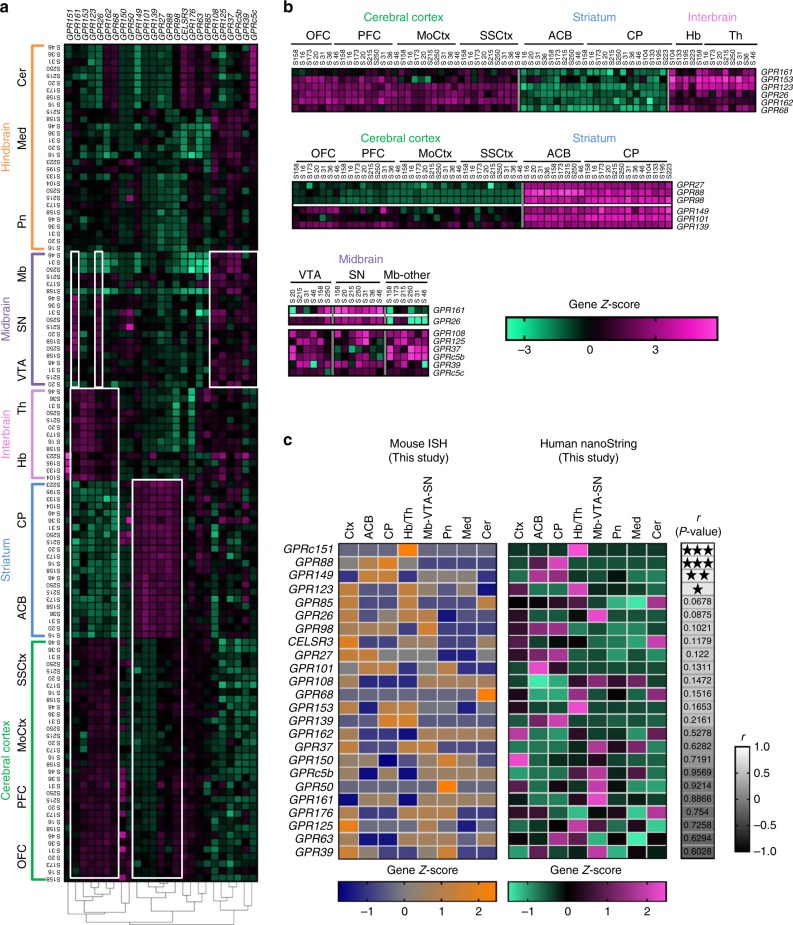
Human expression profiles and cross-species comparison for 25 brain oGPCRs. **a** Hierarchical gene clustering shows expression levels of 25 oGPCRs, determined by nanoString nCounter system, across the 14 human brain regions in samples obtained from 4 to 13 individuals. For each individual subject (S) the sample is indicated by assigned identification numbers for example subject 20 is “S20’. **b** Three panels from cluster (**a**) and outlined in white are extracted here, to illustrate low interindividual variability, and highlight salient features of the cluster. Top, cortical and thalamic (Hb + Th) restricted oGPCRs form a cluster with low to no expression in the striatum. Middle, striatal (ACB and CP) oGPCRs can be subdivided into a striatal/non-cortical cluster or corticostriatal cluster. Bottom, localized (VTA and SN) and widespread oGPCRs in the midbrain. **c** Comparison of mouse (combined DIG- and RNAscope ISH) and human data from this study in eight brain centers. oGPCR distribution in the mouse ISH, (orange (high) to blue (low) mouse, *n* = 2) was correlated to the grouped human nanoString data (magenta (high) to cyan (low), *n* = 4–13). Color bars below indicate expression levels for mouse data (scale 2.48 to −1.78, interval 1) and human data (scale 2.48 to −1.49, interval 1). Clustering shows highly similar (top) to less-well correlated (bottom) oGPCR expression patterns. Pearson correlation coefficients (*r*) and their *P*-values are shown to the right for each oGPCR comparison in a grayscale gradient heatmap from white (positive) to black (negative). Pearson correlation coefficients, scale −1 to 1.0, interval 0.3. This analysis shows 17 positively correlated oGPCRs, among which 4 oGPCRs show significant profile similarity profile, (*Gpr151*, *Gpr88*, *Gpr149* and *Gpr123* (*Adgra1*)) denoted by ***P < 0.001, **P < 0.01, and *P < 0.05. Annotations: Ctx, Cortex; orbital frontal cortex (OFC; BA11), prefrontal cortex (PFC; BA9-10), motor cortex (MoCtx; BA4), somatosensory cortex (SSCtx; BA1,2,3), ACB, nucleus accumbens; CP, caudate putamen; Hb, habenula; thalamus (Th + Hb), Med, Medulla; SN, substantia nigra; Pn, Pons; Mb, midbrain (Mb), VTA, ventral tegmental area; Cer, cerebellum. *Gprc5c* is not shown due to a lack of variation in expression for the selected areas

Hierarchical clustering of genes for individual subject sample RNA counts is displayed in Fig. 5a. A first observation is the high homogeneity of oGPCR expression across individuals within a given region reflecting low interindividual variability, and likely therefore the high quality of the samples. A second observation is the very distinct expression pattern for each oGPCR, as previously observed in the mouse brain. A closer look at the brain areas that govern emotional and cognitive functions revealed several clusters of oGPCRs. For example, a cluster composed of *GPR161*, *GPR153*, *GPR123 (ADGRA1)*, *GPR26, GPR162*, and *GPR68* shows localized cortical and thalamic oGPCRs, with little to no striatal expression (Fig. 5b, top). *GPR27*, *GPR88*, *GPR98 (ADGRV1)*, *GPR101*, *GPR139* and *GPR149* are well detected in both striatal sub-regions (CP and ACB), *GPR101* being higher in ACB, but only *GPR27*, *GPR88*, *GPR98 (ADGRV1)* also show significant cortical expression (Fig. 5b, middle). Finally, in the midbrain, *GPR161* and *GPR26* show expression restricted to VTA and SN, whereas *GPR108*, *GPR125 (ADGRA3)*, *GPR37*, *GPRc5c*, *GPR39* and GPRc5b are widely expressed across midbrain regions (Fig. 5b, bottom).

### Translatability

We finally compared our mouse and human datasets, in order to evaluate translatability of the mouse resource. We combined DIG and RNAscope ISH expression data in the mouse (Fig. 5c, left), and grouped the individual human subject data (Fig. 5c, right), to examine oGPCR expression profiles in eight brain regions including Ctx (OFC, PFC, MoCtx, SSCtx), ACB, CP, Hb + Th, Mb + VTA + SN. *GPRC5C* was excluded from this analysis, due to homogenous expression across all the considered regions in the ISH datasets. For all the oGPCRs, Student’s *t*-test showed no statistical difference across mouse and human distribution profile (Supplementary Table 1). Further, Pearson correlation analysis revealed that 71% oGPCRs were positively correlated, among which 4 (*GPR151*, *GPR88*, *GPR149*, and *GPR123 (ADGRA1)*) showed statistically significant similarity (Pearson correlation, *r* from 0.8067 to 1*, P* values from 0.0155 to <0.0001, also see Supplementary Table 3). Considering potential discordant expression patterns, only 7 oGPCRs (*GPRC5B*, *GPR50*, *GPR*161 *GPR176*, *GPR125 (ADGRA3)*, *GPR63*, and *GPR39*) showed inverse correlation, but none of them reached significance. For the latter oGPCRs, discrepancies in expression profiles between mouse and human brains may arise from genuine distribution across species or to the limited sampling in the human dataset. Overall, the high level of well-correlated oGPCR expression patterns demonstrates great promise for transfer between mouse models to human diseases.

## Discussion

This is the first public brain oGPCR mapping resource to our knowledge. This study reports in-depth anatomical expression analysis in the mouse brain for each oGPCR, extensive comparison of the data with publicly available gene expression databases and, finally, the quantitative expression analysis of selected candidates in samples from the Douglas human brain bank. Although mRNA transcript levels do not necessarily reflect levels of protein expression^72^, any detectable oGPCR in principle can yield an efficient pharmacological target, and the present database is a starting point to predict gene function^73,74^. We anticipate that the combined datasets, all-available at the resource http://ogpcr-neuromap.douglas.qc.ca, will be of valuable use to the neuroscience community in efforts to position oGPCRs based on their expression patterns and their conservation between mouse and human brains.

Our database has characteristics that distinguish this resource from other public information. With regards to oGPCR spatial anatomy in the mouse brain, only two resources are available. GENSAT (5000 genes) is a mouse ISH database focused on developmental gene expression changes across the whole genome (http://www.gensat.org/bgem_ish.jsp), and as of today, contains only 27 brain oGPCRs. Meanwhile, Allen brain (http://mouse.brain-map.org/) has carried out large-scale ISH experiments for about 20,000 genes in the mouse brain, and these include most oGPCRs (except for *Gpr2*7). However, because of the high throughput nature of this massive enterprise, coronal sections are lacking and expression is undetectable for approximately half of oGPCRs. Our study reports 78 oGPCR expression profiles, fine-mapped throughout the mouse brain with optimized probes, and thus definitely characterizes the expression pattern of each oGPCR transcript with high precision. Importantly, our study combines two ISH-based mapping approaches. We used both the classic histochemical detection method (DIG-ISH), which yielded semi-quantifiable datasets (Fig. 2a), and the newer high amplification ISH method RNAscope^44^. Overall, abundant oGPCRs transcripts were reliably detected with both ISH methods, and the two datasets showed consistent patterns based on manual observation (and see http://ogpcr-neuromap.douglas.qc.ca). The low abundant oGPCRs undetectable by DIG-ISH, such as *Gpr139*, *Gpr149*, and *Gpr162*, were easily detected with RNAscope providing expression patterns with cellular resolution for oGPCRs that otherwise remained undetected in large-scale approaches (Supplementary Figure 2).

Key information needed to design oGPCR projects involve determining cell subtypes. We therefore searched an existing cortical RNAseq database^43^ for all brain oGPCRs across 7 brain cell types. Indeed, this cortical centered dataset is only a starting point and oGPCRs may potentially be expressed in different cell types depending on the brain structure. Another critical aspect of this study is the correlation analysis with existing information. Several approaches have been used to map gene expression in the brain^75^, and these involve either ISH-based mapping methods, as performed in this study, or microarray-based technologies applied to microdissected brain regions (see Komatsu et al.^33^, Kasukawa et al.^35^ and http://human.brain-map.org/) that provide quantitative information on gene expression, but limited spatial resolution.

Transcriptome expression data provide information that is fairly distinct in nature from ISH data, but because the information is available, we converted our ISH mapping results into semi-quantitative information to cross-validate mouse data (mouse/mouse) and also initiate cross-species comparison (mouse/human). We found that our data are well aligned with publicly available microarray mouse (BrainStars) and human (Allen Brain) datasets. In fact, ~65% of oGPCRs were detected in both our ISH study and the DNA microarray databases. Further, 48 (80%) and 39 (70%) oGPCRs transcripts showed comparable expression profiles in mouse/mouse (Fig. 3a) and mouse/human (Fig. 3b) comparisons, respectively. This indicates a high degree of consistency for oGPCR expression profiles across multiple detection techniques in the mouse, as well as a high level of conservation from mouse to human (Fig. 3c). We therefore are confident that the database provided by the present study offers a strong basis for oGPCR evaluation in strategic decisions. To our knowledge only one similar study was published, addressing nuclear orphan receptors (Gofflot et al.^74^).

A third unique feature of our database is the inclusion of detailed expression profiles for 25 oGPCRs in the human brain. Selection of the 25 candidates was based on multiple criteria, combining our own experimental data, correlation studies with existing databases, current literature (Supplementary Table 2) and our own interest in brain circuits that govern emotions and cognition, and are possibly involved in addiction and mood disorder pathologies. As human brain bank samples are limited, we employed nanoString, a technology that engineers fluorescent barcoded nucleic acid probes that can be digitally imaged allowing for as many as 800 genes to be probed in a single sample^71^. The nanoString results yielded highly reproducible quantification for the 25 selected oGPCRs, with surprisingly low interindividual variability (Fig. 5a). Of note, the latter experiment identified a top-four oGPCR group with greatest similarity across our own mouse and human data. In this group, *Gpr88*, *Gpr123* (Adgra1), *Gpr149*, and *Gpr151* all show significantly correlated profiles in mouse and human samples. All 4 receptors have in common a primarily neuronal pattern according to our own ISH images observation and a RNA-Seq database from the mouse cortex (Table 1)^43^. Knowledge on these 4 oGPCRs largely varies: *Gpr88* is likely the most studied oGPCR in rodent models^22–26,28,76^ but human data^31^ and reports on drug development^29,30,77^ are still limited; *Gpr151* shows an intriguing localized expression in the habenula, and is virtually undetectable anywhere else in the brain and body^35,78–81^, and this receptor remains entirely open to functional studies and drug discovery; *Gpr149* shows broader distribution in brain and spinal cord with a potential role in sensory processing^82^, but is currently investigated in reproductive biology because of substantial expression in ovaries^83^ *Gpr123* belongs to adhesion GPCRs potentially implicated in brain development, and genome wide association linked this receptor to bipolar disorders^84^. Finally, 7 oGPCRs showed low homology between mouse and human expression patterns, and further studies will be required to determine whether animal models are best appropriate to understand their role in human brain function and disease.

In conclusion, selecting an oGPCR to undertake drug discovery programs is a challenging issue, and predicting which may lead to exploitable targets is difficult. Our study is a step towards this goal and the entire dataset should propel advancement in both oGPCR and brain research.

## Methods

### Animals

Mice were housed in a temperature, humidity controlled animal facility (21 ± 2 °C, 55 ± 10% humidity) on a 12 h dark-light cycle with food and water ad libitum. C57/Bl6J male mice (*n* = 32) aged 10 weeks from Charles River were used. All experiments were performed in accordance with the European Communities Council Directive of 26 May 2010 and approved by the local ethical committee (Com’Eth 2010-003 CREMEAS, 2003-10-08-[1]-58). All efforts were made to minimize the number of animals used and their suffering.

### Mouse tissue preparation

Mice were sacrificed by cervical dislocation, brains were rapidly removed, frozen in OCT (Optimal Cutting Temperature medium, Thermo Scientific) in a freezing mold and stored at −80 °C until use. Coronal brain sections (25 μm) placed onto Superfrost® Plus slides (Thermo Scientific) were obtained using a cryostat (Leica CM3050 S) at −20 °C. Mounted slices were stored at −20 °C until use.

### Riboprobe synthesis

To generate non-radioactive RNA riboprobes, commercially available plasmids were purchased for each orphan GPCR gene (Supplementary Data 1) from Source Bioscience (Nottingham, United Kingdom). Obtained plasmids were amplified and purified using the DNA purification kit, NucleoBond® Xtra Midi (Macherey-Nagel, Germany). Restriction endonuclease digestion reactions were performed on 15 µg of plasmid DNA to linearize the vectors. Restriction enzymes were chosen to obtain a final probe length of 250-800 base pairs. Linearized vectors were purified and then subjected to in vitro transcription of anti-sense ribopobes. One microgram of linearized DNA was transcribed using the appropriate polymerase (Promega, Madison, WI, USA) and concomitantly digoxigenin (DIG)-labeled by the 10× DIG RNA labeling mix (Roche, Germany) according to the manufacturer’s instructions. The resulting Riboprobes were then purified, the concentrations were quantified by spectrophotometry (Nanodrop Labtech ND-1000) and quality was assessed with 1% agarose gel electrophoresis.

### DIG in situ hybridization

Mounted brain slices were fixed with 4% paraformaldehyde (Carlo Erba, Italy) in 1× phosphate buffered saline (PBS) (Sigma-Aldrich) for 10 min, followed by acetylation with acetic anhydride (Sigma-Aldrich) for 10 min with washing in PBS between steps. Afterward, slides were submitted to successive dehydration baths: EtOH 60%, 70, 95, 100%, chloroform, EtOH 100, 95%. After drying the slides, hybridization overnight with 150 ng of probe per slide was carried out at 70 °C. Sections were placed into 5 × SSC solution at room temperature, followed by two washes in 0.2 × SSC, 1 h at 70 °C and 5 min at room temperature. After three washes in Tris/NaCl, blocking in normal goat serum (Sigma) was done at room temperature for 1 h. Anti-DIG antibody (1/2500, Roche, Germany) was added and incubated for 2 h at room temperature followed by 3 washes in Tris/NaCl and exposure with NBT (nitroblue tetrazolium, Roche, Germany) and BCIP (5-bromo-4-chloro-3-indolyl phosphate, toluidinium salt Roche, Germany) color substrates. After washes in water and drying, slides are mounted with Pertex (Microm, France) and stored at room temperature. Image acquisition was performed with the slide scanner NanoZoomer 2 HT (Hamamatsu, Shizuoka, Japan) all the analysis was done on NDP View software (Hamamatsu, Shizuoka, Japan). Control probes were included in each experiment. Negative controls were treated with hybridization buffer lacking probes and probes for *Oprm1*, *Penk*, or *Gpr88* were included as positive controls (Supplementary Fig. 1a, *upper panel*).

### RNAscope® in situ hybridization

A high amplification system single molecule detection ISH method, RNAscope® (Advanced Cell Diagnostics (ACD), Hayward, California), was used for ultrasensitive detection and visualization of weakly expressed mRNA, in brain tissue prepared with the same methods as tissue used for DIG labeled riboprobe ISH (see above). All mouse specific probes were synthesized by the manufacturer. Positive (mouse *Ppib*) and negative (*DapB*) control probes were included in each experiment (Supplementary Fig. 1a, lower panel). RNAscope experiments were performed according to the manufacturer’s instructions for fresh frozen sections. Briefly, sections were fixed in 4% formaldehyde in 1× PBS overnight at 4 °C and dehydrated in successive 3 min baths of ethanol (60, 75, 95, 100%) and chloroform. After drying, two steps of pretreatment were performed, including a 16-min step of protease digestion. Hybridization with specific probes was then performed for 2 h at 40 °C, followed by six steps of amplification. Two washes of 2 min were observed between each amplification step. Fast Red was used as a chromogen for the exposure step, which was monitored from 10 to 25 min at room temperature under microscopic control. A counterstain included with the kit was used in ISH early-on but obstructed distinguishing the red oGPCR stain from the counterstain, (Supplementary Fig. 1b, lower panel *Gpr88*) and was removed for subsequent experiments. After washing in water and drying, slides were mounted with Ecomount (Biocare Medical, Concord, CA, USA) and stored at room temperature. Image acquisition was performed with a slide scanner NanoZoomer 2 HT (Hamamatsu, Shizuoka, Japan) all the analysis was done on NDP View software (Hamamatsu, Shizuoka, Japan).

### ISH scoring and public database comparative analysis

DIG-ISH and RNAscope mapping analysis was adapted from the classification of GenePaint annotation procedures (http://www.genepaint.org/) and previously described^74^. Manual annotation of expression across brain regions, identified on the basis of published brain atlas, Allen Brain Atlas (ABA)^37^, of expression are defined: 3.5 as strong with color precipitate completely filling the cells, 2.5 as moderate detection with color precipitate filling half of cell, 1.5 as weak detection and 0.5 as no detectable level above background, (Supplementary Fig. 1b, upper DIG-ISH and lower RNAscope). All images were scored by two independent observers. Final scoring was the compilation of the two independent scores. Multiple probe sets per oGPCR, if any, were averaged before further analysis. As labeling intensities may differ between probes, scoring was performed based on relative intensities across all brain sections for each probe. DIG-ISH resulting scores were submitted to hierarchical cluster analysis for gene axis with Euclidean distance and average linkage using TreeView and Cluster 3 software^85^.

Group comparison analysis was performed with the mouse DNA microarray data from the BrainStars database (http://brainstars.org/)^35^ included 48 punched regions compiled into 12 regions to match our analyzed regions for 60 oGPCRs. Analysis of human ABA complete normalized microarray datasets were compiled from six subjects and 106 brain regions were merged into 15 brain regions (http://human.brain-map.org/static/download) for 56 oGPCRs. Regions left out of correlations were due to a lack of corresponding regions in datasets and categorization of sub-nuclei were according to ABA classification. In the event of a gene having multiple probe sets data were first averaged, followed by region and donor averaging. To facilitate group comparison, the datasets were first converted into gene *Z*-scores (regional expression is expressed in terms of standard deviations (SD) from the mean of each gene [*Z*-score = (oGPCR region—mean of oGPCR regions)/SD of oGPCR regions].

To determine oGPCR cell pattern in the brain, we searched the Brain RNA-Seq database (https://web.stanford.edu/group/barres_lab/brain_rnaseq.html)^43^ for all 92 oGPCRs (Table 1). Shown is the category of cell subtype (neurons, astrocytes, microglia, endothelial cells, pericytes, or various maturation states of oligodendrocytes) followed by oGPCRs with the highest Fragments Per Kilobase of transcript per Million (FPKM) mapped reads. If FPKM were below 1.0, virtually undetectable was written.

To compare the mouse ISH public databases for the selected 25 oGPCR subgroups, gene z-scores were computed for each gene per technique. All of the selected oGPCRs were available in the qPCR dataset obtained from Regard et al. supplementary files but in only 7 of 11 regions. 14 oGPCRs were not found and 1 was undetectable in adult GENSAT-ISH dataset. The ten selected oGPCRs found at GENSAT are available only as images. Thus, we used the same criteria that we applied in our study’s ISH mapping to semi-quantify the GENSAT dataset and converted them to *Z*-scores. Allen brain mouse ISH data for all but 3 oGPCRs (*Gpr139*, *Gpr153*, and *Gpr27*) was obtained as “Raw expression values” from the website and converted into gene *Z*-scores for the 11 regions. Finally, DIG-ISH quantification from this study (Fig. 1) was converted into gene *Z*-scores for the 11 regions. *Gpr63*, *Gpr139*, *Gpr149*, and *Gprc5c* were compared using this study’s RNAscope ISH quantification.

### Human brain tissue dissections

Postmortem (PM delay 6–24 h) tissues from 14 brain regions of 4–13 (dependent on region availability) male adult individuals were obtained from the Suicide section of the Douglas – Bell Canada Brain Bank (Douglas Mental Health University Institute, Montreal, Quebec, Canada). The subjects had died suddenly from accidental or natural causes and were aged 20–55. Dissections were performed on fresh frozen 0.5 cm-thick coronal sections with the guidance of a human brain atlas^86^. Samples were prepared from the following regions: orbital frontal cortex (OFC; BA11), prefrontal cortex (PFC; BA9-10), motor cortex (MoCtx; BA4), somatosensory cortex (SSCtx; BA1,2,3), nucleus accumbens (ACB), caudate putamen (CP), habenula (Hb), thalamus (Th + Hb), Medulla (Med), substantia nigra (SN), Pons (Pn) midbrain (Mb), ventral tegmental area (VTA), and cerebellum (Cer). Ethical approval (Protocol 15/04) for this study was obtained from the Institutional Review Board of the Douglas Mental Health University Institute.

### Human RNA preparation and integrity analysis

Human total RNA was isolated using NucleoZol (Macherey-Nagel, Düren, Germany). A NanoDrop ND2000 (ThermoFisher, Waltham, MA, USA) spectrophotometer was used to determine RNA quantity and quality. RNA integrity numbers (RIN) were measured by automated electrophoresis with the 2200 TapeStation system (Agilent, Santa Clara, CA, USA). Low RNA concentrations and integrity (RIN < 4) were excluded from analysis. RINs higher than 5 were considered good quality and samples with a RIN higher than 8 were considered perfect^87^.

### NanoString

Experiments were performed at the Jewish General Hospital Molecular Pathology Center (Montréal, QC, Canada) using NanoString nCounter targeted gene expression profiling as described previously^88^. In brief 5 µl of 20 ng/µl total RNA was hybridized with the reporter and capture probes at 65 °C for overnight. Probes were custom designed to target 25 oGPCRs and 5 housekeeping genes as internal controls: *GAPDH*, *ACTB*, *HPRT1*, *RPL19*, *RPL0*. The samples were then processed with the nCounter Prep Station to purify the hybridized targets and affix them to the cartridge for imaging with a CCD camera. Barcodes were counted for each target molecule. The data were analyzed using the nSolver version 3.02 (nanoString Technologies). Non-specific binding was subtracted by measuring binding densities of negative control ERCC RNA probes that target genes not expressed in human tissues. Positive control normalization parameters were followed as indicated by the manufacturer. Housekeeping genes used for normalization was dependent on low coefficient of variance (%CV). Finally, *Z*-scores for each gene were used to facilitate comparison with the other datasets.

### Data analysis

For all correlations, datasets were converted to *Z*-scores. ISH scores, BrainStars microarray, Allen Brain microarray, and normalized nanoString data were first converted into gene Z-scores (regional expression is expressed in terms of standard deviations (SD) from the mean of each gene [*Z*-score = (oGPCR region—mean of oGPCR regions)/SD of oGPCR regions]. Statistical analyses were carried out using Prism 6.0 or 7.0 and heatmaps were done with Prism 7.0 software (GraphPad Software, Inc). When the *P*-value was less than 0.05 data was considered as statistically significant.

### Data availability

Data used in this study were retrieved from RNA Riboprobe accession numbers GenBank NCBI (https://www.ncbi.nlm.nih.gov/genbank/), the Brain RNA Seq website (https://web.stanford.edu/group/barres_lab/brain_rnaseq.html), the BrainStars database (http://brainstars.org/), and the Allen Institute human gene expression database (http://human.brain-map.org/). The mouse ISH datasets generated and analyzed in this study are freely available at (http://ogpcr-neuromap.douglas.qc.ca). The human nanoString data generated and analyzed in this study are freely available to download at (http://ogpcr-neuromap.douglas.qc.ca).

## Electronic supplementary material

Supplementary Information

Description of additional Supplementary Infomation

Supplementary Data 1

Supplementary Data 2

## References

[1] Harding SD (2018). The IUPHAR/BPS Guide to pharmacology in 2018: updates and expansion to encompass the new guide to immunopharmacology. Nucleic Acids Res.

[2] Ellis C, Smith A (2004). Highlighting the pitfalls and possibilities of drug research. Nat. Rev. Drug Discov..

[3] Hauser AS, Attwood MM, Rask-Andersen M, Schioth HB, Gloriam DE (2017). Trends in GPCR drug discovery: new agents, targets and indications. Nat. Rev. Drug Discov..

[4] Wacker D, Stevens RC, Roth BL (2017). How ligands illuminate GPCR molecular pharmacology. Cell.

[5] Davenport AP (2013). International Union of Basic and Clinical Pharmacology. LXXXVIII. G protein-coupled receptor list: recommendations for new pairings with cognate ligands. Pharmacol. Rev..

[6] Nagata K, Katayama Y, Sato T, Kwon Y, Kawabata T (2016). Toward the next step in G protein-coupled receptor research: a knowledge-driven analysis for the next potential targets in drug discovery. J. Struct. Funct. Genom..

[7] Regard JB, Sato IT, Coughlin SR (2008). Anatomical profiling of G protein-coupled receptor expression. Cell.

[8] Uhlen M (2015). Proteomics. Tissue-based map of the human proteome. Science.

[9] Fricker, L. D. & Devi, L. A. Orphan neuropeptides and receptors: novel therapeutic targets. *Pharmacol. Ther*. 10.1016/j.pharmthera.2017.11.006 (2017). 10.1016/j.pharmthera.2017.11.006 PMC5899030 29174650

[10] Komatsu H (2015). Novel therapeutic GPCRs for psychiatric disorders. Int J. Mol. Sci..

[11] Alavi MS, Shamsizadeh A, Azhdari-Zarmehri H, Roohbakhsh A (2017). Orphan G protein-coupled receptors: the role in CNS disorders. Biomed. Pharmacother..

[12] Manglik A (2016). Structure-based discovery of opioid analgesics with reduced side effects. Nature.

[13] Caprioli, D., Justinova, Z., Venniro, M. & Shaham, Y. Effect of novel allosteric modulators of metabotropic glutamate receptors on drug self-administration and relapse: a review of preclinical studies and their clinical implications. *Biol. Psychiatry*. 10.1016/j.biopsych.2017.08.018 (2017). 10.1016/j.biopsych.2017.08.018 PMC5837933 29102027

[14] McCorvy, J. D. et al. Structure-inspired design of beta-arrestin-biased ligands for aminergic GPCRs. *Nat*. *Chem*. *Biol*. 10.1038/nchembio.2527 (2017). 10.1038/nchembio.2527 PMC5771956 29227473

[15] Roth BL, Kroeze WK (2015). Integrated approaches for genome-wide interrogation of the druggable non-olfactory G protein-coupled receptor superfamily. J. Biol. Chem..

[16] Ngo T (2017). Orphan receptor ligand discovery by pickpocketing pharmacological neighbors. Nat. Chem. Biol..

[17] Khan, M. Z. & He, L. Neuro-psychopharmacological perspective of Orphan receptors of Rhodopsin (class A) family of G protein-coupled receptors. *Psychopharmacology*. 10.1007/s00213-017-4586-9 (2017). 10.1007/s00213-017-4586-9 28289782

[18] Becker JA (2008). Transcriptome analysis identifies genes with enriched expression in the mouse central extended amygdala. Neuroscience.

[19] Conti B (2007). Region-specific transcriptional changes following the three antidepressant treatments electro convulsive therapy, sleep deprivation and fluoxetine. Mol. Psychiatry.

[20] Massart R, Guilloux JP, Mignon V, Sokoloff P, Diaz J (2009). Striatal GPR88 expression is confined to the whole projection neuron population and is regulated by dopaminergic and glutamatergic afferents. Eur. J. Neurosci..

[21] Massart R (2016). Developmental and adult expression patterns of the G-protein-coupled receptor GPR88 in the rat: Establishment of a dual nuclear-cytoplasmic localization. J. Comp. Neurol..

[22] Logue SF (2009). The orphan GPCR, GPR88, modulates function of the striatal dopamine system: a possible therapeutic target for psychiatric disorders?. Mol. Cell Neurosci..

[23] Meirsman AC, de Kerchove drsquoExaerdeA, Kieffer BL, Ouagazzal AM (2017). GPR88 in A2A receptor-expressing neurons modulates locomotor response to dopamine agonists but not sensorimotor gating. Eur. J. Neurosci..

[24] Meirsman AC (2016). Mice lacking GPR88 show motor deficit, improved spatial learning, and low anxiety reversed by delta opioid antagonist. Biol. Psychiatry.

[25] Meirsman, A. C., Robe, A., de Kerchove d’Exaerde, A. & Kieffer, B. L. GPR88 in A2AR neurons enhances anxiety-like behaviors. *eNeuro*. 10.1523/ENEURO.0202-16.2016 (2016). 10.1523/ENEURO.0202-16.2016 PMC4987659 27570825

[26] Quintana A (2012). Lack of GPR88 enhances medium spiny neuron activity and alters motor- and cue-dependent behaviors. Nat. Neurosci..

[27] Ehrlich, A. T. et al. Mapping GPR88-venus illuminates a novel role for GPR88 in sensory processing. *Brain Struct*. *Funct*. 10.1007/s00429-017-1547-3 (2017). 10.1007/s00429-017-1547-3 PMC5871604 29110094

[28] Arefin, T. et al. Remodeling of sensorimotor brain connectivity in Gpr88 deficient mice. *Brain Connect*. 10.1089/brain.2017.0486 (2017). 10.1089/brain.2017.0486 PMC5653097 28882062

[29] Jin C, Decker AM, Langston TL (2017). Design, synthesis and pharmacological evaluation of 4-hydroxyphenylglycine and 4-hydroxyphenylglycinol derivatives as GPR88 agonists. Bioorg. Med. Chem..

[30] Jin C, Decker AM, Harris DL, Blough BE (2016). Effect of substitution on the aniline moiety of the GPR88 agonist 2-PCCA: synthesis, structure-activity relationships, and molecular modeling studies. ACS Chem. Neurosci..

[31] Alkufri F, Shaag A, Abu-Libdeh B, Elpeleg O (2016). Deleterious mutation in GPR88 is associated with chorea, speech delay, and learning disabilities. Neurol. Genet..

[32] Carecchio M, Mencacci NE (2017). Emerging monogenic complex hyperkinetic disorders. Curr. Neurol. Neurosci. Rep..

[33] Komatsu H (2014). Anatomical transcriptome of G protein-coupled receptors leads to the identification of a novel therapeutic candidate GPR52 for psychiatric disorders. PLoS One.

[34] Nishiyama, K. et al. FTBMT, a novel and selective GPR52 agonist, demonstrates antipsychotic-like and procognitive effects in rodents revealing a potential therapeutic agent for schizophrenia. *J*. *Pharmacol*. *Exp*. *Ther*. 10.1124/jpet.117.242925 (2017). 10.1124/jpet.117.242925 28851764

[35] Kasukawa T (2011). Quantitative expression profile of distinct functional regions in the adult mouse brain. PLoS One.

[36] Hawrylycz MJ (2012). An anatomically comprehensive atlas of the adult human brain transcriptome. Nature.

[37] Lein ES (2007). Genome-wide atlas of gene expression in the adult mouse brain. Nature.

[38] Magdaleno S (2006). BGEM: an in situ hybridization database of gene expression in the embryonic and adult mouse nervous system. PLoS Biol..

[39] Gloriam DE, Fredriksson R, Schioth HB (2007). The G protein-coupled receptor subset of the rat genome. BMC Genom..

[40] Hamann J (2015). International Union of Basic and Clinical Pharmacology. XCIV. Adhesion G protein-coupled receptors. Pharmacol. Rev..

[41] Sharman JL (2011). IUPHAR-DB: new receptors and tools for easy searching and visualization of pharmacological data. Nucleic Acids Res..

[42] Ghate A (2007). Identification of novel striatal genes by expression profiling in adult mouse brain. Neuroscience.

[43] Zhang Y (2014). An RNA-sequencing transcriptome and splicing database of glia, neurons, and vascular cells of the cerebral cortex. J. Neurosci..

[44] Wang F (2012). RNAscope: a novel in situ RNA analysis platform for formalin-fixed, paraffin-embedded tissues. J. Mol. Diagn..

[45] Hu H (2016). Reward and aversion. Annu Rev. Neurosci..

[46] Russo SJ, Nestler EJ (2013). The brain reward circuitry in mood disorders. Nat. Rev. Neurosci..

[47] Koob GF, Volkow ND (2016). Neurobiology of addiction: a neurocircuitry analysis. Lancet Psychiatry.

[48] Bailey MR, Simpson EH, Balsam PD (2016). Neural substrates underlying effort, time, and risk-based decision making in motivated behavior. Neurobiol. Learn Mem..

[49] Kringelbach ML (2005). The human orbitofrontal cortex: linking reward to hedonic experience. Nat. Rev. Neurosci..

[50] Volkow ND, Baler RD (2015). NOW vs LATER brain circuits: implications for obesity and addiction. Trends Neurosci..

[51] Price JL, Drevets WC (2012). Neural circuits underlying the pathophysiology of mood disorders. Trends Cogn. Sci..

[52] Belin D, Everitt BJ (2008). Cocaine seeking habits depend upon dopamine-dependent serial connectivity linking the ventral with the dorsal striatum. Neuron.

[53] Kalivas PW, O’Brien C (2008). Drug addiction as a pathology of staged neuroplasticity. Neuropsychopharmacology.

[54] Mitsi V, Zachariou V (2016). Modulation of pain, nociception, and analgesia by the brain reward center. Neuroscience.

[55] Yang H (2018). Nucleus accumbens subnuclei regulate motivated behavior via direct inhibition and disinhibition of VTA dopamine subpopulations. Neuron.

[56] Proulx CD, Hikosaka O, Malinow R (2014). Reward processing by the lateral habenula in normal and depressive behaviors. Nat. Neurosci..

[57] Bromberg-Martin ES, Matsumoto M, Hikosaka O (2010). Dopamine in motivational control: rewarding, aversive, and alerting. Neuron.

[58] Boulos LJ, Darcq E, Kieffer BL (2017). Translating the Habenula-from rodents to humans. Biol. Psychiatry.

[59] Viswanath H, Carter AQ, Baldwin PR, Molfese DL, Salas R (2013). The medial habenula: still neglected. Front Hum. Neurosci..

[60] Sanders J, Nemeroff C (2016). The CRF system as a therapeutic target for neuropsychiatric disorders. Trends Pharmacol. Sci..

[61] Namburi P (2015). A circuit mechanism for differentiating positive and negative associations. Nature.

[62] Sharp BM (2017). Basolateral amygdala and stress-induced hyperexcitability affect motivated behaviors and addiction. Transl. Psychiatry.

[63] Kim J, Zhang X, Muralidhar S, LeBlanc SA, Tonegawa S (2017). Basolateral to central amygdala neural circuits for appetitive behaviors. Neuron.

[64] Gilpin NW, Herman MA, Roberto M (2015). The central amygdala as an integrative hub for anxiety and alcohol use disorders. Biol. Psychiatry.

[65] Drui G (2014). Loss of dopaminergic nigrostriatal neurons accounts for the motivational and affective deficits in Parkinson’s disease. Mol. Psychiatry.

[66] Bjorklund A, Dunnett SB (2007). Dopamine neuron systems in the brain: an update. Trends Neurosci..

[67] Damier P, Hirsch EC, Agid Y, Graybiel AM (1999). The substantia nigra of the human brain. II. Patterns of loss of dopamine-containing neurons in Parkinson’s disease. Brain.

[68] Rub U (2014). Huntington’s disease (HD): degeneration of select nuclei, widespread occurrence of neuronal nuclear and axonal inclusions in the brainstem. Brain Pathol..

[69] Lucki I (1998). The spectrum of behaviors influenced by serotonin. Biol. Psychiatry.

[70] Li Y (2016). Serotonin neurons in the dorsal raphe nucleus encode reward signals. Nat. Commun..

[71] Veldman-Jones MH (2015). Evaluating robustness and sensitivity of the nanostring technologies nCounter platform to enable multiplexed gene expression analysis of clinical samples. Cancer Res..

[72] Carlyle BC (2017). A multiregional proteomic survey of the postnatal human brain. Nat. Neurosci..

[73] Zhang W (2004). The functional landscape of mouse gene expression. J. Biol..

[74] Gofflot F (2007). Systematic gene expression mapping clusters nuclear receptors according to their function in the brain. Cell.

[75] Gaspar I, Ephrussi A (2015). Strength in numbers: quantitative single-molecule RNA detection assays. Wiley Interdiscip. Rev. Dev. Biol..

[76] Ben Hamida, S. et al. Increased alcohol seeking in mice lacking Gpr88 involves dysfunctional mesocorticolimbic networks. *Biol. Psychiatry*10.1016/j.biopsych.2018.01.026 (2018). 10.1016/j.biopsych.2018.01.026 PMC6054571 29580570

[77] Dzierba CD (2015). Design, synthesis, and evaluation of phenylglycinols and phenyl amines as agonists of GPR88. Bioorg. Med. Chem. Lett..

[78] Broms J (2017). Monosynaptic retrograde tracing of neurons expressing the G-protein coupled receptor Gpr151 in the mouse brain. J. Comp. Neurol..

[79] Broms J, Antolin-Fontes B, Tingstrom A, Ibanez-Tallon I (2015). Conserved expression of the GPR151 receptor in habenular axonal projections of vertebrates. J. Comp. Neurol..

[80] Ignatov A, Hermans-Borgmeyer I, Schaller HC (2004). Cloning and characterization of a novel G-protein-coupled receptor with homology to galanin receptors. Neuropharmacology.

[81] Kobayashi Y (2013). Genetic dissection of medial habenula-interpeduncular nucleus pathway function in mice. Front. Behav. Neurosci..

[82] Manteniotis S (2013). Comprehensive RNA-Seq expression analysis of sensory ganglia with a focus on ion channels and GPCRs in Trigeminal ganglia. PLoS One.

[83] Edson MA, Lin YN, Matzuk MM (2010). Deletion of the novel oocyte-enriched gene, Gpr149, leads to increased fertility in mice. Endocrinology.

[84] Ament SA (2015). Rare variants in neuronal excitability genes influence risk for bipolar disorder. Proc. Natl Acad. Sci. USA.

[85] Saeed AI (2003). TM4: a free, open-source system for microarray data management and analysis. Biotechniques.

[86] Mai, J. K., Voss, T. & Paxinos, G. *Atlas of the Human Brain*. 3rd edn, (Elsevier/Academic Press, 2008).

[87] Fleige S, Pfaffl MW (2006). RNA integrity and the effect on the real-time qRT-PCR performance. Mol. Asp. Med..

[88] M’Boutchou MN, van Kempen LC (2016). Analysis of the tumor microenvironment transcriptome via nanostring mRNA and miRNA expression profiling. Methods Mol. Biol..

