# Supplementary material for: Expression map of 78 brain-expressed mouse orphan GPCRs provides a translational resource for neuropsychiatric research
Source: Commun Biol. 2018 Aug 6;1:102. doi: 10.1038/s42003-018-0106-7 (PMC6123746; doi:10.1038/s42003-018-0106-7)
Supplement: Supplementary file 1 — Supplementary Information [file 42003_2018_106_MOESM1_ESM.pdf]

SUPPLEMENTARY INFORMATION

SUPPLEMENTARY FIGURES

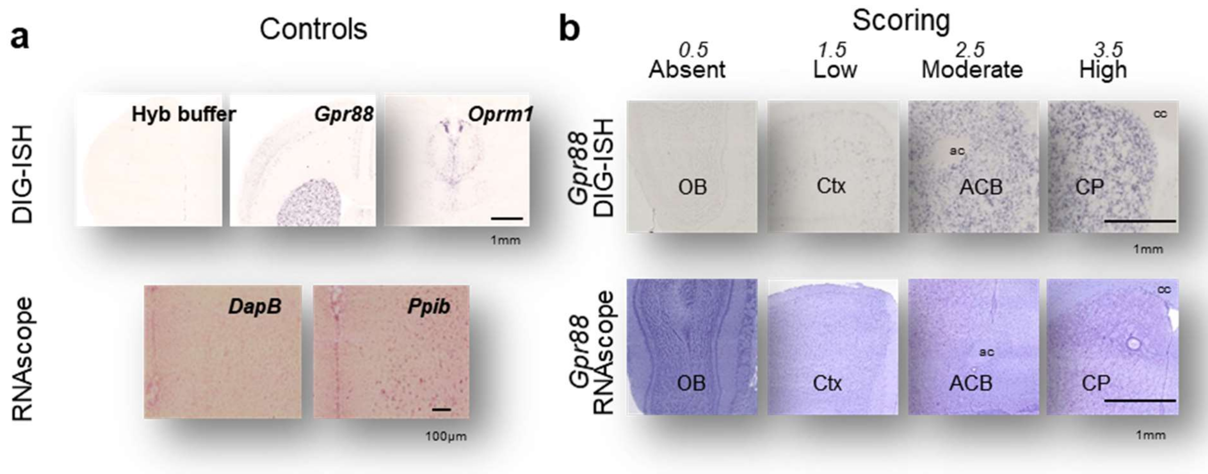

Supplementary Figure 1 | Controls and four levels of scored expression. a, Representative controls used in each experiment for both DIG and RNAscope ISH methods. *Upper* panel in blue shows DIG-ISH controls, negative - hybridization buffer without probe, positive - *Gpr88* enriched in caudate putamen (CP) and *Oprm1* enriched expression in medial habenula and paraventricular thalamus, scale bars = 1 mm. *Lower* panel in red shows RNAscope ISH, negative control, *DapB*, is not detected in CP whereas positive control *Ppib* is detected throughout the brain shown here in the prefrontal cortex, scale bar = 100 µm. **b**, Representative images from ISH scoring demonstrates *Gpr88* is highly expressed in CP, moderately in nucleus accumbens (ACB) but lowly expressed in the cortex (Ctx) and absent in the olfactory bulb (OB). Both methods detected *Gpr88* similarly, *upper row,* DIG-ISH and *lower* row shows RNAscope (*Gpr88*-red, counter stain-blue) images. Scale bar is 1mm. Annotations: anterior commissure (ac), corpus callosum (cc).

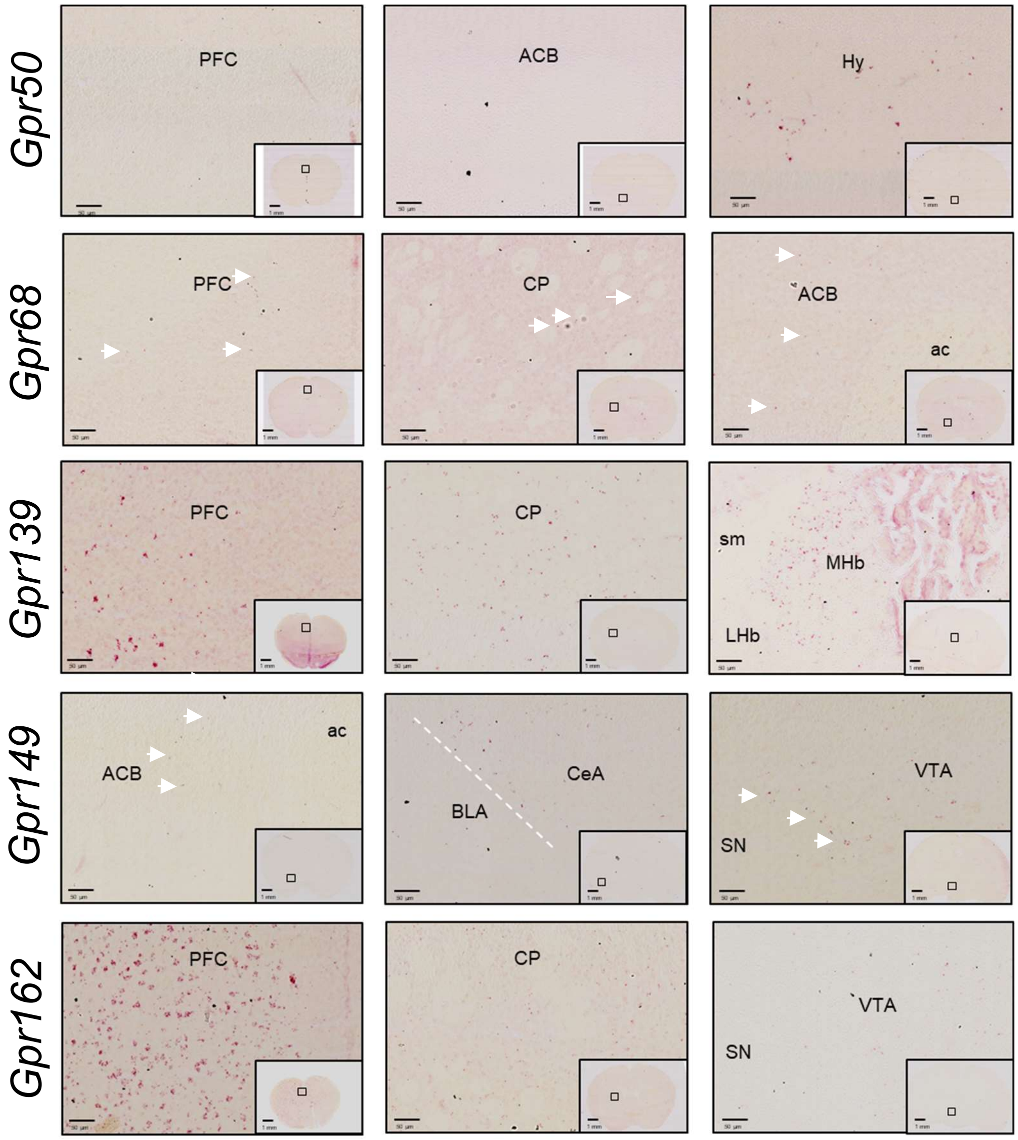

Supplementary Figure 2 | RNAscope ISH reveals low abundant oGPCRs. Shown here are representative images of oGPCRs lowly detected by DIG-ISH and revealed by RNAscope ISH. (*Top to Bottom*) *Gpr50* is absent in prefrontal cortex (PFC) and nucleus accumbens (ACB) but enriched in hypothalamus (Hy). *Gpr68* is sparsely localized to PFC and caudate putamen (CP) and ACB. *Gpr139* is high in PFC moderate in CP and high in the medial habenula (MHb). *Gpr149* is moderate in ACB and central extended amygdala (CeA) in contrast to basal lateral amygdala (BLA) and moderate in ventral tegmental area (VTA). Finally, *Gpr162* is high in PFC, moderate in CP and VTA. 1.25x insets are of whole slice view and boxed region corresponds to 20x image, scale bar for the former is 1 mm and the latter is 50 µm. White arrows demonstrate sparse labeling pattern for *Gpr68* and *Gpr149*. Annotations: anterior commissure (ac), stria medullaris (sm), lateral habenula (LHb), substantia nigra (SN).

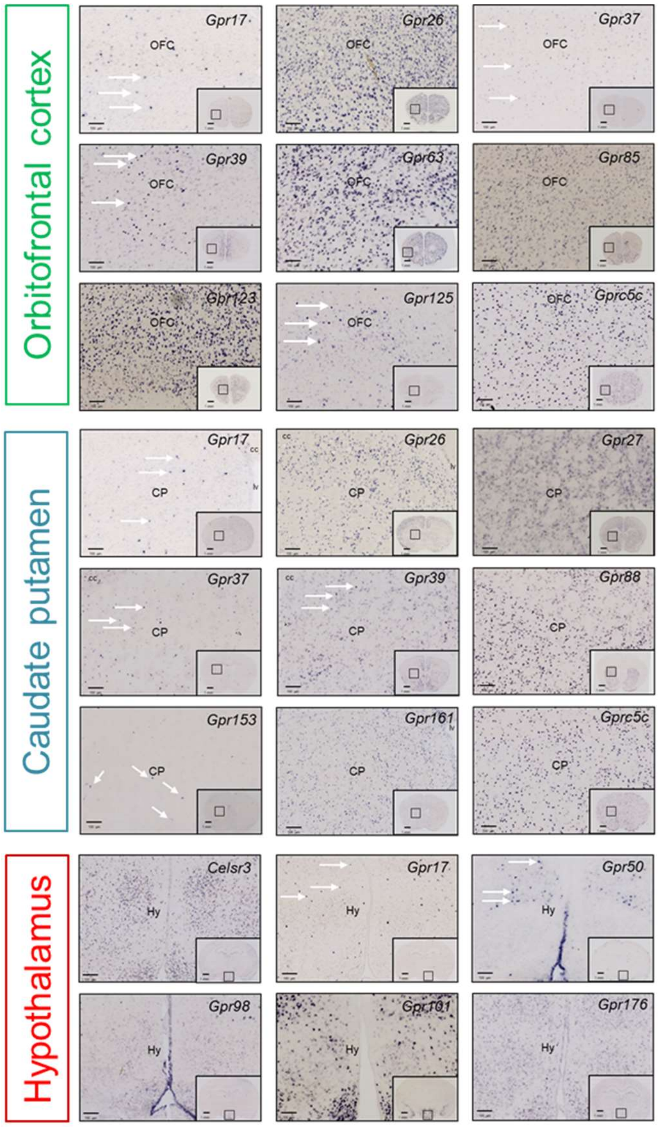

Supplementary Figure 3 | oGPCRs with expression in orbitofrontal cortex, caudate putamen and hypothalamus. Shown here are additional panels of 25 oGPCRs in brain centers relevant to drug abuse and mood disorders (**Fig. 4 overview**). DIG-ISH oGPCR expression in mouse orbitofrontal cortical (OFC) areas, important for reward learning and inhibitory control. *Top* panel shows from left to right, considerable expression for *Gpr17*, *Gpr26*, *Gpr37*, *Gpr39*, *Gpr63*, *Gpr85*, *Gpr123 (Adgra1), Gpr125 (Adgra3)* and *Gprc5c*. oGPCRs in striatal areas are regarded for neural outputs and inputs involved in movement, motivation and habit. *Middle* panel shows from left to right, *Gpr17*, *Gpr26*, *Gpr27*, *Gpr37*, *Gpr39*, *Gpr88*, *Gpr153*, *Gpr161* and *Gprc5c* in the caudate putamen (CP). *Bottom* panel shows oGPCRs with remarkable expression in another reward behavior area, hypothalamus (Hy) (from left to right, *Celsr3 (Adgrc3), Gpr17, Gpr50, Gpr98, Gpr101, and Gpr176*). The 1.25x insets are approximately Allen brain atlas (ABA) #32-37 (OFC), ABA #43-47 (CP) and #68-72 (Hy). The boxed region of the 1.25x insets corresponds to magnified area of 10x image, scale bar for the former is 1 mm and the latter is 100 μm. White arrows demonstrate sparse DIG labeling pattern for *Gpr17, Gpr37, Gpr39, Gpr50, Gpr125 (Adgra3), Gpr153* and *Gpr176*. Annotations, anterior commissure (ac), lateral ventricle (lv), shell (sh), corpus callosum (cc).

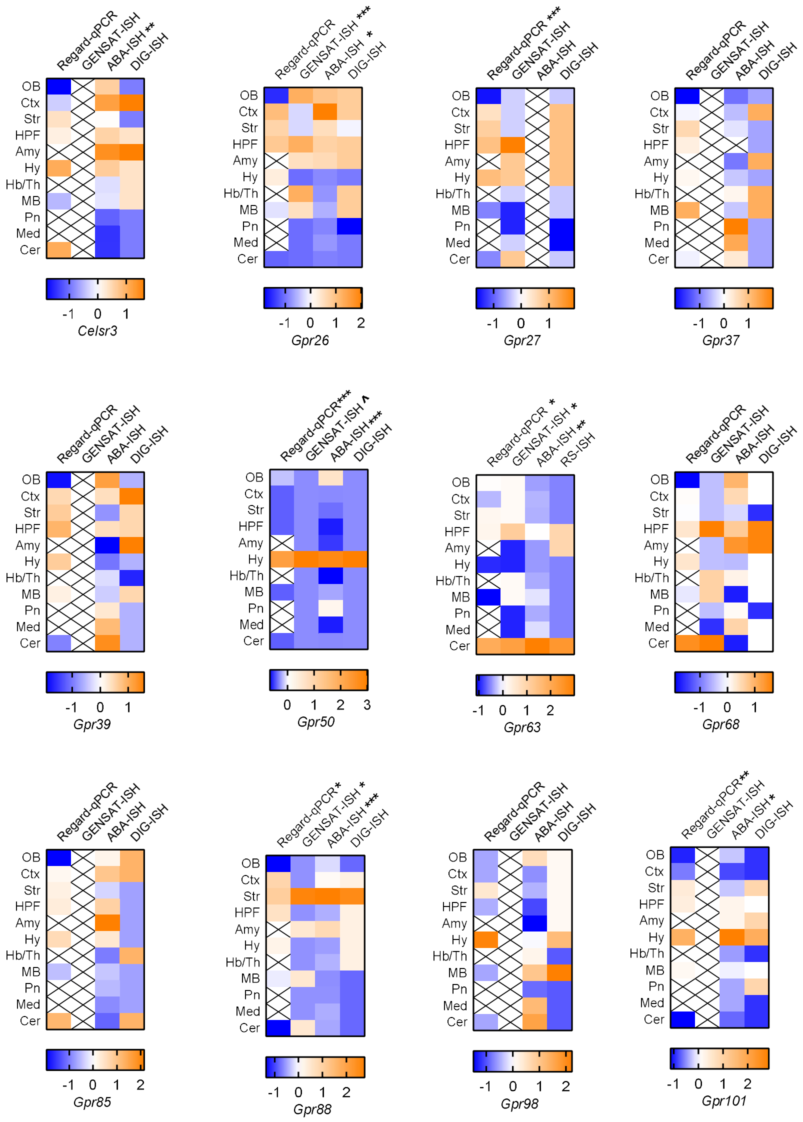

Supplementary Figure 4 | Heat maps for cross-comparison of datasets from 25 selected oGPCRs with 3 mouse public datasets. To aid a collective understanding of brain oGPCR expression we compared ISH in this study, either (DIG or RNAscope/RS), to 25 oGPCRs x 7 regions in Regard-qPCR, 10 oGPCRS x 11 regions in GENSAT-ISH and 22 oGPCRs x 11 regions in ABA-ISH. To compare the different datasets, gene z-scores were computed for the brain regions (*see methods*). The scale below each heatmap indicates the level of gene expression with intervals of 1, low (*blue*) to high (*orange*) or data unavailable (*cross-hatch*). For each gene, significance is shown as **P*<0.05, ***P*<0.01 and ****P*<0.001 if Pearson correlation analysis reveals positive correlation between public dataset with our ISH (DIG or RS) datasets. "^" indicates correlation is a perfect line. Annotations- olfactory bulb (OB), cortex (Ctx), striatum (Str), hippocampus (HPF), amygdala (Amy), hypothalamus (Hy), habenula and thalamus (Hb/Th), midbrain (MB), pons (Pn), medulla (Med) and cerebellum (Cer).

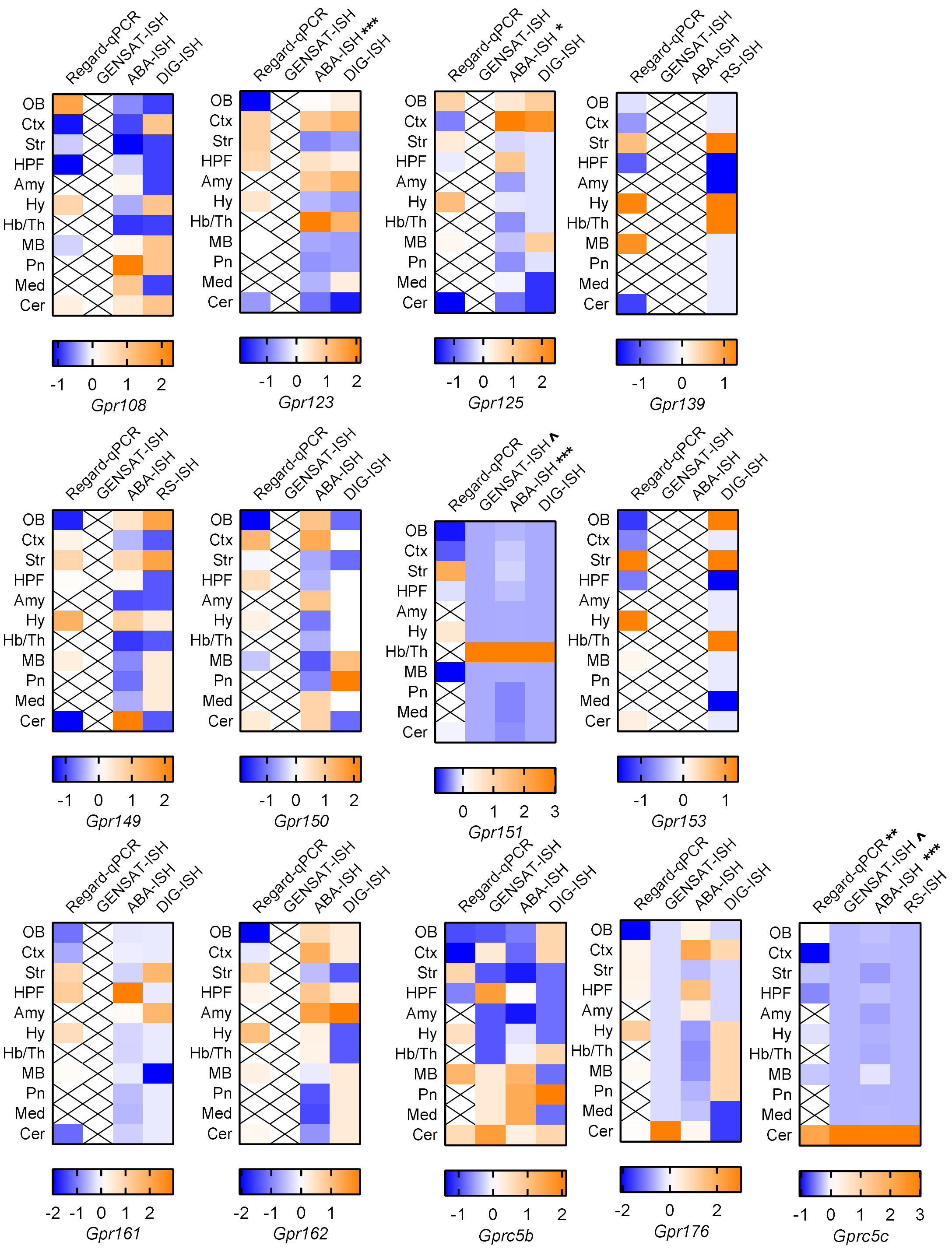

Supplementary Figure 4 | continued

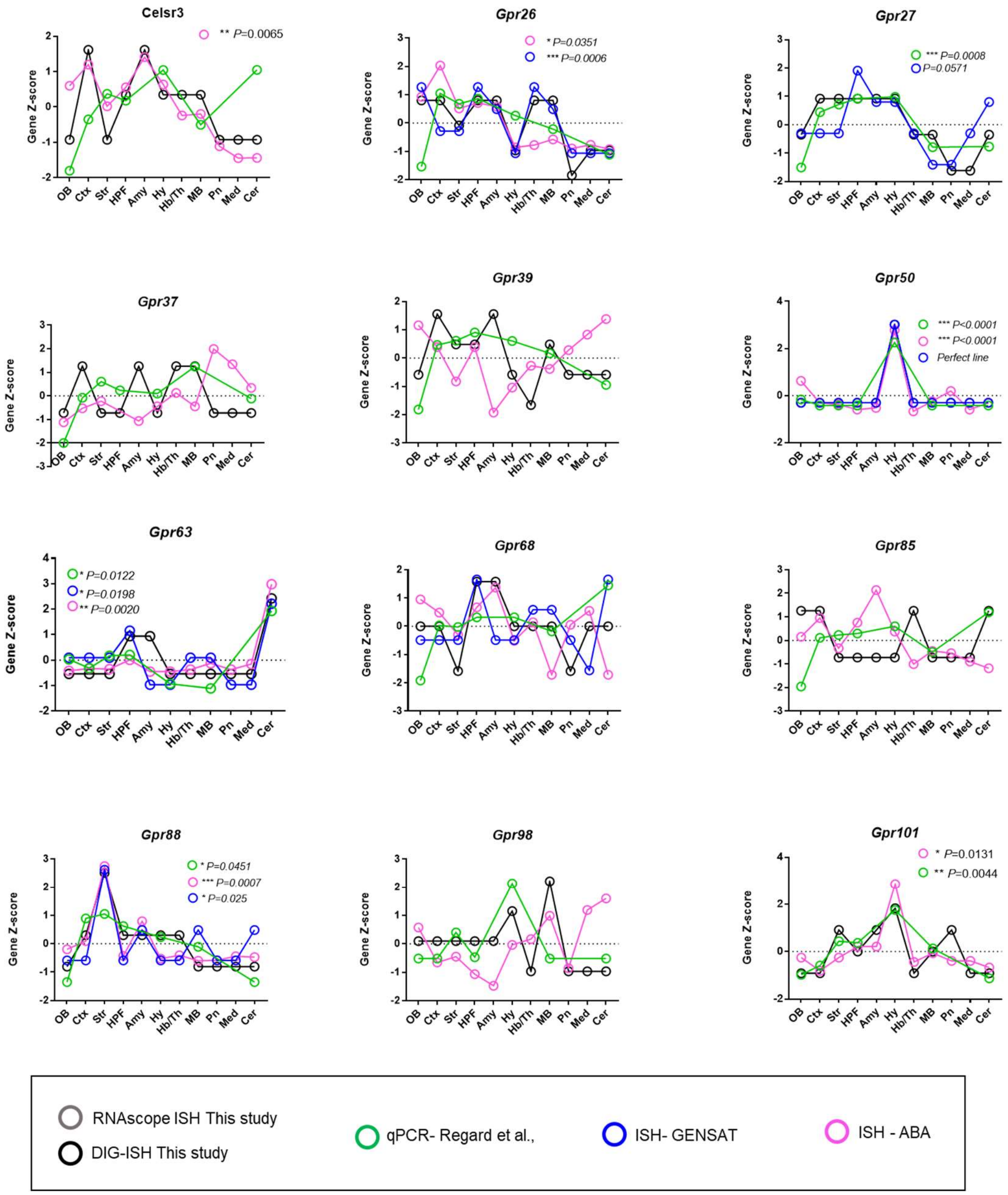

Supplementary Figure 5 | Line graphs for cross-comparison of datasets from 25 selected oGPCRs with 3 mouse public datasets. We compared ISH in this study (DIG or RNAscope/RS) to 25 oGPCRs x 7 regions in Regard-qPCR, 10 oGPCRS x 11 regions in GENSAT-ISH and 22 oGPCRs x 11 regions in ABA-ISH. To compare the different datasets, gene z-scores were computed (*see methods*). Datasets are shown as qPCR (green), ISH-GENSAT (blue), ISH-ABA (magenta) or our datasets DIG-ISH (black), RNAscope-ISH (gray). If Pearson correlation analysis revealed positively correlated datasets compared to our ISH (DIG or RS), significance is shown as **P*<0.05, ***P*<0.01 and ****P*<0.001. “Perfect line” indicate datasets having a perfect linear correlation. Annotations- olfactory bulb (OB), cortex (Ctx), striatum (Str), hippocampus (HPF), amygdala (Amy), hypothalamus (Hy), habenula and thalamus (Hb/Th), midbrain (MB), pons (Pn), medulla (Med) and cerebellum (Cer).

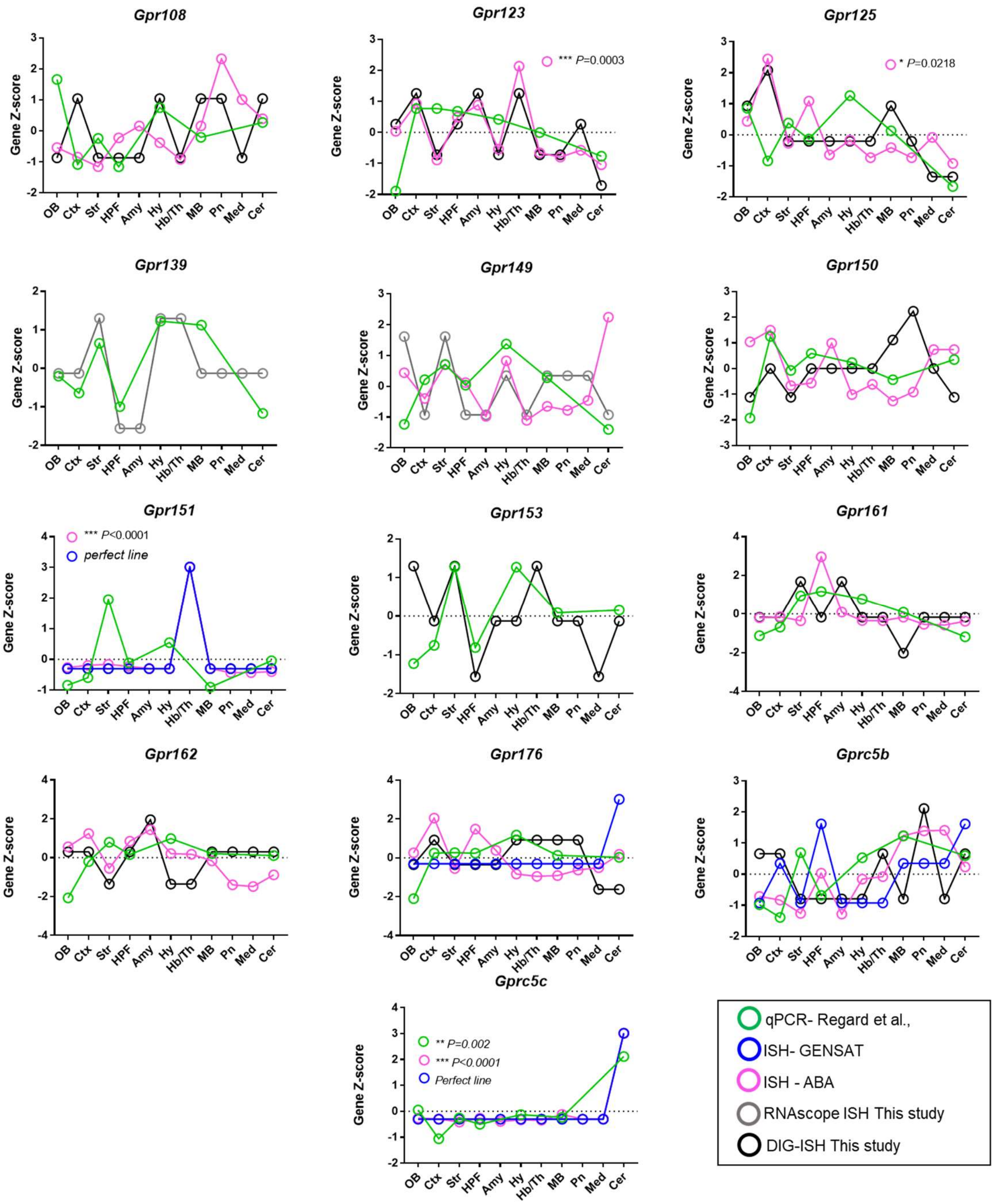

Supplementary Figure 5 | continued

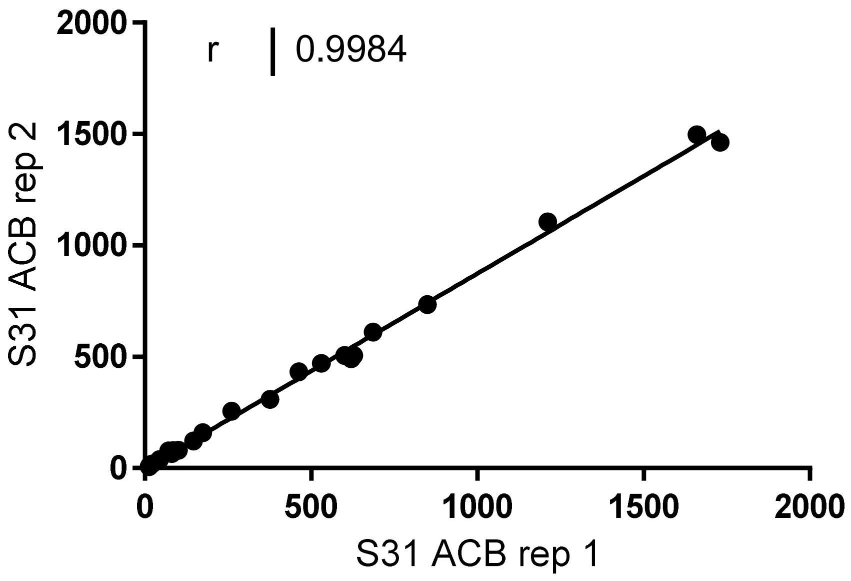

Supplementary Figure 6 | nanoString technical replicates are highly reproducible. Representative technical replicate (rep) from the nanoString assay. Subject 31 (S-31) nucleus accumbens (ACB) sample was run in duplicate and we obtained a highly similar result between these technical replicates. Pearson correlation demonstrates positive correlation between sample runs with r = 0.9984.

SUPPLEMENTARY TABLES

Supplementary Table 1 | Student's *t*-test for group comparisons of brain oGPCRs. There was no statistical difference in oGPCR profiles for any of the comparisons. *Column I*, all mouse DIG-ISH vs. mouse DNA microarray (BrainStars, **Figure 3a**). *Column II*, mouse DIG-ISH vs. human DNA microarray (Allen brain, **Figure 3b**). *Column III*, mouse ISH vs. human nanoString (**Figure 5**). $P<0.05$ was considered as statistically significant. NA indicates data were unavailable.

Gene	Mouse DIG-ISH (This study) vs. Mouse microarray BrainStars	Mouse DIG-ISH (This study) vs. Human microarray Allen Brain	Mouse ISH (This study) vs. Human nanoString (This study)
Bai1	1.0000	1.0000	NA
Bai2	1.0000	1.0000	NA
Bai3	1.0000	1.0000	NA
Celsr1	1.0000	1.0000	NA
Celsr2	1.0000	1.0000	NA
Celsr3	1.0000	1.0000	1.0000
Eltd1	1.0000	1.0000	NA
Emr1	1.0000	1.0000	NA
Fpr1	1.0000	1.0000	NA
Gpr1	1.0000	1.0000	NA
Gpr101	1.0000	1.0000	1.0000
Gpr107	1.0000	1.0000	NA
Gpr108	1.0000	1.0000	1.0000
Gpr116	1.0000	1.0000	NA
Gpr123	1.0000	1.0000	1.0000
Gpr125	1.0000	1.0000	1.0000
Gpr135	1.0000	1.0000	NA
Gpr139	NA	NA	1.0000
Gpr149	1.0000	1.0000	1.0000
Gpr15	1.0000	1.0000	NA
Gpr150	1.0000	1.0000	1.0000
Gpr151	1.0000	1.0000	1.0000
Gpr153	1.0000	1.0000	1.0000
Gpr155	1.0000	1.0000	NA
Gpr161	1.0000	1.0000	1.0000
Gpr162	1.0000	1.0000	1.0000
Gpr165	1.0000	NA	NA
Gpr171	1.0000	1.0000	NA
Gpr173	1.0000	1.0000	NA
Gpr175	1.0000	NA	NA
Gpr176	1.0000	1.0000	1.0000
Gpr182	1.0000	1.0000	NA
Gpr19	1.0000	1.0000	NA
Gpr2	1.0000	0.2554	NA
Gpr22	1.0000	1.0000	NA

Supplementary Table 1 | Continued

Gene	Mouse DIG-ISH (This study) vs. Mouse microarray BrainStars	Mouse DIG-ISH (This study) vs. Human microarray Allen Brain	Mouse ISH (This study) vs. Human nanoString (This study)
Gpr26	1.0000	1.0000	1.0000
Gpr27	1.0000	1.0000	1.0000
Gpr3	1.0000	1.0000	NA
Gpr30	1.0000	NA	NA
Gpr37	1.0000	1.0000	1.0000
Gpr39	1.0000	1.0000	1.0000
Gpr45	1.0000	1.0000	NA
Gpr48	1.0000	1.0000	NA
Gpr49	1.0000	1.0000	NA
Gpr50	1.0000	1.0000	1.0000
Gpr56	1.0000	1.0000	NA
Gpr61	1.0000	1.0000	NA
Gpr63	1.0000	1.0000	1.0000
Gpr64	1.0000	1.0000	NA
Gpr68	1.0000	0.0000	1.0000
Gpr75	1.0000	1.0000	NA
Gpr85	1.0000	1.0000	1.0000
Gpr88	1.0000	1.0000	1.0000
Gpr98	1.0000	1.0000	1.0000
Gprc5b	1.0000	1.0000	1.0000
Gprc5c	1.0000	NA	NA
Lphn2	1.0000	1.0000	NA
Lphn3	1.0000	1.0000	NA
Mchr1	1.0000	1.0000	NA
Mrge	1.0000	1.0000	NA
Smoh	1.0000	1.0000	NA

Supplementary Table 2 | Selection of a 25 oGPCRs. Evaluation of expression patterns for the selection of a 25 oGPCR subgroup. oGPCRs were first subdivided by class [1-4] and then sorted according to column ranking from left to right. Criteria evaluated to select 25 oGPCRs: **Column 1** – shows whether the global distribution pattern was localized (L), widespread (W) or not detected (N) with DIG-ISH, **Column 2** - (Mouse BrainStars) Pearson correlation coefficients (*r*) between DIG-ISH and mouse DNA microarray BrainStars (see **Figure 3a**; NA indicates data were unavailable), **Column 3** - Pearson correlation coefficients (*r*) between DIG-ISH and human DNA microarray Allen Brain (see **Figure 3b**; NA indicates data were unavailable), **Column 4** - shows the number of PubMed entries for each receptor. A lower number of entries was desirable, indicating understudied oGPCRs. 25 oGPCRs were selected for human study (gold filled cell). Level of interest as target oGPCR is indicated by colors: Low (blue), Moderate (white), High (red).

	Gene	Global pattern	Mouse BrainStars	Human Allen brain	PubMed
Class A	*Gpr151*	L	0.9961	0.9964	6
	Gpr50	L	0.9496	-0.1297	52
	Gpr88	L	0.9393	0.7529	39
	Gpr39	L	0.9088	0.0422	136
	Gpr101	L	0.8312	0.6558	31
	Gpr176	L	0.7980	0.2248	5
	Gpr27	L	0.7813	0.3639	14
	Gpr153	L	0.6549	0.1587	7
	Gpr48	L	0.6121	-0.3824	32
	Gpr22	L	0.5983	0.7935	15
	Gpr26	L	0.5788	0.4476	16
	Gpr63	L	0.4925	0.1466	9
	Gpr150	L	0.4472	0.3499	5
	Gpr162	L	0.3810	0.4749	7
	Gpr68	L	0.3358	0.4583	76
	Gpr173	L	0.2257	0.4921	10
	Gpr165	L	0.1966	NA	2
	Gpr49	L	0.0708	-0.0866	22
	Gpr37	L	0.0112	0.1989	68
	Gpr75	L	-0.3133	-0.4662	12
	Gpr45	L	-0.3837	-0.1498	6
	Pgr15l	L	NA	NA	0
	Gpr21	L	NA	NA	10
	Gpr85	W	0.4135	0.0163	19
	Gpr161	W	0.0946	-0.6064	29
	Gpr30	W	0.0465	NA	711
	Mrge	W	-0.1373	-0.5020	6
	Gpr171	W	-0.3576	0.3323	11
	Gpr83	W	NA	NA	31
	Gpr17	W	NA	NA	87
	Gpr135	N	0.4602	0.4222	7
	Gpr182	N	0.3991	-0.2001	3
	Mchr1	N	0.3798	0.0669	263
	Gpr15	N	0.2344	-0.1148	104
	Gpr61	N	0.2319	0.1242	12
	Gpr1	N	0.1587	-0.4010	158
	Gpr2	N	-0.0198	-0.0477	15
	Gpr149	N	-0.0663	0.0802	4
	Fpr1	N	-0.3375	0.0195	287
	Gpr3	N	-0.3432	0.0064	71
	Gpr19	N	-0.4672	-0.2131	13
	Taar9	N	NA	NA	3

Supplementary Table 2 | continued

	Gene	Global pattern	Mouse BrainStars	Human Allen brain	PubMed
Class A	*Gpr82*	N	NA	NA	4
	Taar4	N	NA	NA	7
	Gpr146	N	NA	NA	10
	Taar6	N	NA	NA	17
	Gpr139	N	NA	NA	19
	Gpr87	N	NA	NA	23
	Gpr12	N	NA	NA	39
	Gpr183	N	NA	NA	51
	Mas1	N	NA	NA	76
Class B	*Bai1 (Adgrb1)*	L	0.8175	0.4499	91
	Gpr56 (Adgrg1)	L	0.6777	0.3165	139
	Gpr123 (Adgra1)	L	0.6573	0.7557	8
	Gpr98 (Adgrv1)	L	0.4944	0.1622	67
	Lphn2 (Adgrl2)	L	0.2169	0.0649	19
	Gpr125 (Adgra3)	L	0.1943	0.0069	35
	Celsr2 (Adgrc2)	L	0.1547	0.3609	88
	Bai3 (Adgrb3)	L	0.0442	0.0643	32
	Gpr116 (Adgrf5)	L	0.0257	-0.4308	25
	Celsr1 (Adgrc1)	L	0.0015	0.2860	99
	Bai2 (Adgrb2)	W	0.7325	0.7538	31
	Celsr3 (Adgrc3)	W	0.0710	0.3987	62
	Lphn3 (Adgrl3)	N	0.4245	0.3620	47
	Gpr64 (Adgrg2)	N	0.3898	-0.1682	26
	Eltd1 (Adgrl4)	N	0.1618	-0.3448	27
	Emr1 (Adgre1)	N	-0.0572	-0.1037	98
	Gpr111 (Adgrf2)	N	NA	NA	5
	Gpr124 (Adgra2)	N	NA	NA	37
Class C	*Gprc5b*	L	0.4194	0.1273	29
	Gprc5c	W	0.1201	NA	13
	Gpr51	N	NA	NA	5
Others	*Gpr175*	L	0.3598	NA	4
	Gpr155	L	0.3112	0.6016	8
	Gpr108	L	0.2383	0.2970	3
	Smoh	L	-0.3170	-0.1216	75
	Gpr137b	L	NA	NA	13
	Gpr107	N	-0.4528	0.2560	10

Supplementary Table 3 | Pearson correlation statistics

**Fig. 3a**	***r***	***P* values**	**95%CI low**	**95%CI high**
Gpr151	0.9961	0.0000	0.9856	0.9989
Gpr50	0.9496	0.0000	0.8256	0.9861
Gpr88	0.9393	0.0000	0.7926	0.9832
Gpr39	0.9088	0.0000	0.6999	0.9744
Gpr101	0.8312	0.0008	0.4919	0.9513
Bai1	0.8175	0.0012	0.4588	0.9471
Gpr176	0.798	0.0019	0.4134	0.9410
Gpr27	0.7813	0.0027	0.3759	0.9357
Bai2	0.7325	0.0067	0.2735	0.9197
Gpr56	0.6777	0.0155	0.1698	0.9011
Gpr123	0.6573	0.0202	0.1340	0.8940
Gpr153	0.6549	0.0208	0.1297	0.8931
Gpr48	0.6121	0.0344	0.0589	0.8777
Gpr22	0.5983	0.0399	0.0372	0.8726
Gpr26	0.5788	0.0486	0.0073	0.8653

**Fig. 3b**	***r***	***P* values**	**95%CI low**	**95%CI high**
GPR151	0.9964	0	0.9889	0.9988
GPR22	0.7935	0.0004	0.4738	0.9284
GPR123	0.7557	0.0011	0.3972	0.9141
BAI2	0.7538	0.0012	0.3935	0.9134
GPR88	0.7529	0.0012	0.3918	0.9130
GPR101	0.6558	0.0079	0.2162	0.8743
GPR155	0.6016	0.0177	0.1291	0.8514
GPR161	-0.6064	0.0165	-0.8535	-0.1366

**Fig. 5c**	***r***	***P* values**	**95%CI low**	**95%CI high**
GPR151	1	0	0.9998	1.0000
GPR88	0.9208	0.0012	0.6153	0.9858
GPR149	0.8539	0.007	0.3745	0.9731
GPR123	0.8067	0.0155	0.2365	0.9636

SUPPLEMENTARY REFERENCES

1 Davenport, A. P. *et al.* International Union of Basic and Clinical Pharmacology. LXXXVIII. G protein-coupled receptor list: recommendations for new pairings with cognate ligands. *Pharmacol Rev* **65**, 967-986, doi:10.1124/pr.112.007179 (2013).

2 Gloriam, D. E., Fredriksson, R. & Schiöth, H. B. The G protein-coupled receptor subset of the rat genome. *BMC Genomics* **8**, doi:10.1186/1471-2164-8-338 (2007).

3 Hamann, J. *et al.* International Union of Basic and Clinical Pharmacology. XCIV. Adhesion G protein-coupled receptors. *Pharmacol Rev* **67**, 338-367, doi:10.1124/pr.114.009647 (2015).

4 Sharman, J. L. *et al.* IUPHAR-DB: new receptors and tools for easy searching and visualization of pharmacological data. *Nucleic Acids Res* **39**, D534-538, doi:10.1093/nar/gkq1062 (2011).
