# Supplementary material for: Expression map of 78 brain-expressed mouse orphan GPCRs provides a translational resource for neuropsychiatric research
Source: Commun Biol. 2018 Aug 6;1:102. doi: 10.1038/s42003-018-0106-7 (PMC6123746; doi:10.1038/s42003-018-0106-7)
Supplement: Supplementary file 2 — Description of additional Supplementary Infomation [file 42003_2018_106_MOESM2_ESM.docx]

**Supplementary Data 1** | **Non-radioactive RNA Riboprobes**

Underlined oGPCR probes were cut to length indicated in base pair (bp) column. Purple oGPCRs indicate genes which were further analyzed in human nanoString study.

**Supplementary Data 2 | Regional distribution of oGPCR scoring by DIG-ISH**

oGPCR expression shown by region as high (3.5), moderate (2.5), low (1.5) or absent (0.5) according to DIG-ISH scoring (see **Fig. 2b**).
